# Copper and Zinc Levels in Commercial Marine Fish from Setiu, East Coast of Peninsular Malaysia

**DOI:** 10.3390/toxics10020052

**Published:** 2022-01-24

**Authors:** Chee Kong Yap, Khalid Awadh Al-Mutairi

**Affiliations:** 1 Department of Biology, Faculty of Science, Universiti Putra Malaysia (UPM), Serdang 43400, Selangor, Malaysia; 2 Department of Biology, Faculty of Science, University of Tabuk, Tabuk P.O. Box 741, Saudi Arabia; kmutairi@ut.edu.sa

**Keywords:** commercial fishes, health risks, fish consumption, Cu, Zn

## Abstract

Potentially toxic metals (PTMs) in edible marine fish have been widely reported from at least 15 different regions or countries in the literature. This evidently demonstrates the importance of monitoring the PTMs in fish fillets from a human health risk (HHR) point of view. This study aims to assess the HHR of Cu and Zn in 19 species of marine fish from popular marine fish loading sites at Setiu in Terengganu, on the east coast of Peninsular Malaysia, collected between August 2016 and February 2017. With overall ranges of concentrations (mg/kg dry weight) of Cu (1.50–7.83), and Zn (24.1–80.5), the 19 species of marine fishes from Setiu are good sources of these essential elements because they are below the maximum permissible limits set by seafood safety guidelines. The target hazard quotient values of Cu and Zn were lower than one, indicating non-carcinogenic risks of Cu and Zn in fish consumption. It was also found that the calculated values of the estimated weekly intake were below the established provisional tolerable weekly intake of Cu and Zn. It can be concluded that the consumption of fish from Setiu would not pose adverse effects from the PTMs to consumers. Nonetheless, continuous monitoring is necessary to ensure the safety of consumers who rely heavily on marine fish in Setiu coastal waters.

## 1. Introduction

Among all the papers reviewed on the metals in edible fish, copper (Cu) and zinc (Zn) were usually determined and reported in the literature. This could be attributed to the fact that these are common essential elements with significant health benefits but yet potentially toxic metals (PTMs) when the dietary intake of these two metals is over the thresholds that could potentially pose a human health risk (HHR) [1]. The toxicological aspects of Cu and Zn are well documented by Dorsey et al. [2] and Roney et al. [3], respectively. The environmental health criteria of Cu and Zn are also indicated by World Health Organization (WHO) [4,5]. This demonstrates that Cu and Zn have been paid much attention in terms of their risks to environmental and human health.

Cu is required for iron utilization and as a cofactor for enzymes involved in glucose metabolism and the synthesis of hemoglobin, connective tissue, and phospholipids [6,7,8,9,10]. Extremely high quantities of Cu, on the other hand, can induce acute poisoning. Intentional eating of significant amounts of Cu sulphate has been reported to result in human mortality. Thus, Cu concentrations were set at a safe level by several regulatory organizations. Cu from marine fish is an important source for human health; although very high amounts of Cu intake from marine fish might cause health concerns, such as liver and kidney damage, it is not carcinogenic to humans or animals [7,11].

Zn is found in almost every cell and a wide variety of foods. The essential role of Zn is based on its role as an integral part of several metalloenzymes and as a catalyst for regulating the activity of specific Zn-depended enzymes [10,12]. Zn is also necessary for aquatic organisms, such as fish; however, when Zn reaches its maximum value, it becomes poisonous. Many scientists believe that dietary Zn is the primary cause of elevated Zn in marine fish [13]. High levels of Zn can also harm the pancreas, disrupt protein metabolism, and lead to arteriosclerosis. Humans’ immune systems (lower lymphocyte stimulation response) and cholesterol metabolism will be harmed if they regularly have a high Zn diet [9,14].

In light of the HHA of PTMs in edible marine fish, the PTMs concentration in fish fillets or muscles must consequently be monitored to ensure adherence to food safety rules for consumer protection purposes [15,16]. Despite their many nutritional benefits, such as being high in protein, low in saturated fat, and high in omega fatty acids [17], PTMs may be highly bioaccumulated in the fish body. Hence, the nutritional benefits of fish will be nullified if they are contaminated with PTMs [18].

Many studies have been conducted on the concentrations of PTMs in fish in many regions or countries, such as the Red Sea [19,20,21], India [22,23,24], Pakistan [14,25,26,27,28], Indonesia [29,30], Persian Gulf or Iran [31,32,33,34,35], Bangladesh [36,37,38], Tanzania [39], Turkey [40], China [41,42,43], Mediterranean Sea [44,45], Aegean Sea/Eastern Mediterranean [46,47], Mexico [48], the Black Sea (Bulgaria), and the Ionian Sea (Italy) [49].

Papers published before 2000 are more likely to reflect monitoring data and mostly direct comparisons to maximum permissible limits (MPL) of food safety guidelines of PTMs were reported. Many recent publications on the HHR of PTMs in marine fish were based on the provisional tolerable weekly intake (PTWI) and target hazard quotient (THQ) of the PTMs. For example, Babji et al. [50] reported the levels of four PTMs, including Cu and Zn, in six species of marine fishes in Peninsular Malaysia and compared them to MPLs of the PTMs of food standard guidelines. Kureishy et al. [22] reported levels of six metals, including Cu and Zn, in marine fishes from the Andaman Sea, which was a monitoring study without comparison with any seafood safety guidelines. In more recent publications, besides reporting the PTMs levels, Fathi et al. [51] reported the estimated weekly and daily intakes for four PTMs, including Cu and Zn, which were far below the PTWI limits based on three marine fish collected from Mersing on the east coast of Peninsular Malaysia. Besides reporting that Zn surpassed the different food safety guidelines, Jahangir Sarker et al. [38] also reported THQ values <1.0 for all fish species, indicated the absence of public health hazards. Alipour et al. [52] reported that adult consumers and children in Malaysia and Bangladesh were at considerable non-carcinogenic risk.

Because Malaysians consume about 60–70% of protein from marine fish [53], several monitoring studies of PTMs in the marine fish of Malaysia have been reported in the literature [15,16,50,51,54,55,56,57,58,59,60,61,62]. For example, Agusa et al. [54] determined the concentrations of 21 trace elements (including Cu and Zn) in 12 species of marine fish collected from coastal areas in Malaysia. They reported that seven PTMs, including Cu and Zn, were higher in bigeye scads from Peninsular Malaysia’s east coast than those from the west. Based on nine heavy metals in 46 species of marine fish from the coastal waters of Peninsular Malaysia, Wan Azmi et al. [58] reported that the risk assessment demonstrated THQ values that were lower than one in all fish species, indicating low non-carcinogenic risk and considered safe for human consumption. Salam et al. [59] concluded that local consumers of Kedah and Selangor will face high chronic risk if they consume the popular torpedo scad (*Megalaspis cordyla*) on a regular basis in their diet, based on the THQ value.

However, information on the HHA of PTMs on commercial marine fish on the east coast of Peninsular Malaysia is still lacking. Therefore, the objective of this study is to assess the HHRs of Cu and Zn in 19 species of commercial marine fish collected from two fishing loading sites in Setiu, Terengganu.

## 2. Materials and Methods

### 2.1. Sample Collection

Setiu is one the busiest fishing loading sites and biggest marine fish-based food productions on the east coast of PM, where fishermen capture marine organisms for diet and economic use. As Setiu is facing the urbanization and development of several industries, Setiu coastal marine resources could have received pollution stress [16].

**Figure 1:**
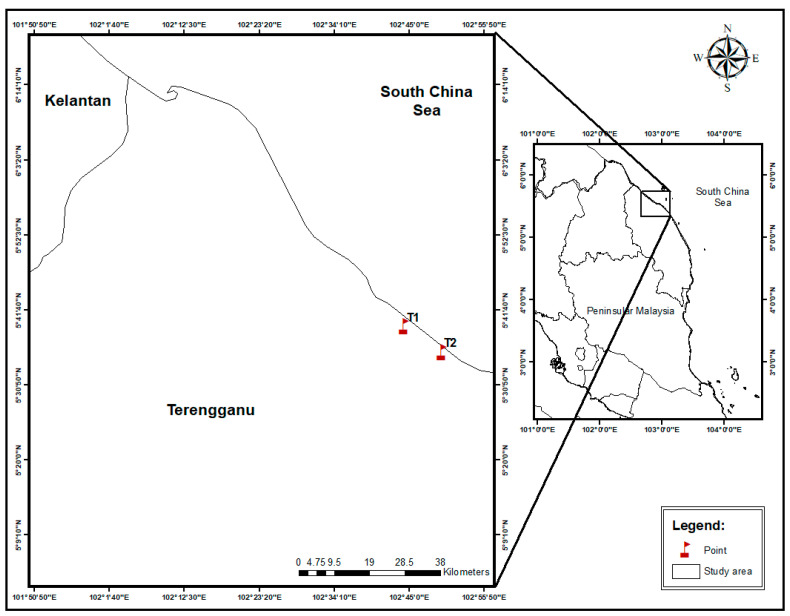
Map of sample collection sites at fishing loading sites in Kampong Fikri (T1) and Kampung Rhu Sepuluh (T2) in Setiu, Terengganu.

In this study, 19 species of commercial marine fishes were collected from two fishing loading sites (Kampong Fikri; 5°39′19″ N, 102°44′16″ E) and Kampung Rhu Sepuluh; 5°35′36″ N, 102°49′42″ E) in Setiu, Terengganu (Figure 1; Table S1). The estimated distance between the sites was about 10 km. The fishes were collected between August 2016 and February 2017 directly from fishermen upon landing at the landing site. For all species, the fish with similar lengths and weights were collected. The fish were classified based on information obtained from www.fishbase.org (assessed on 1 August 2017) and key identifications by Mohsin and Ambak [63] and Matsunuma et al. [64]. The fish sample identification was also cross-checked based on the online data (https://www.fishbase.in/search.php, assessed on 18 December 2021) to ensure the species name, family, and niche habitat of each fish species.

### 2.2. Sample Preparation

To keep the samples fresh, they were placed in a refrigerated box with ice just after they were collected. Ice was employed to prevent tissue deterioration and keep the environment wet during shipment. The samples were then rinsed with water to eliminate any foreign particles, and excess water was drained from the fish using a paper towel. Each fish was then weighed with a computerized electronic balance and its length was measured using a ruler. The length of the fish was measured from the upper jaw’s snout to the tail’s end. The dorsal muscles of the fish were then dissected. Each fish had 10–20 g of dorsal muscle removed. According to Rahman et al. [36], the fish muscles are the primary storage location for metals.

The samples were frozen and delivered to the University Putra Malaysia laboratory after being preserved in a freezer according to their species to avoid cross-contamination. The samples were then frozen in a freezer until metal analysis could be performed. The samples are defrosted at room temperature in the laboratory. Each species was broken up into little pieces and combined to form a composite sample. The muscles were then dried in an oven for 72 h at 60 °C until they reached a constant weight. The goal of drying is to remove excess water and determine the moisture content of the fish, which is important for converting to a wet weight (ww) basis. The weight loss following the drying process was used to determine the moisture content. The samples were then homogenized by grinding them using an agate pestle and mortar. Until further investigation, the sample powder was kept in an airtight plastic bag.

### 2.3. Metal Analysis

In the digestion tube, 5 mL of concentrated nitric acid (HNO_3_; AnalaR grade, BDH 69%) was introduced after 0.50 g of homogenized dried sample was correctly weighed. They were then placed in a hot block digester for an hour at 40 °C for a pre-digestion purpose. Later, the temperature was increased to 140 °C for three hours to fully digest the samples [65,66].

After the digestion was finished, the solution was allowed to cool for 30 min before being diluted with distilled water to a total amount of 40 mL. The acid digest was then filtered through filter paper into acid-washed pillboxes (Whatman no 1).

An air-acetylene flame atomic absorption spectrophotometer (FAAS) was used to determine the amounts of Cu and Zn in the digested fish samples. The detection limits of the FAAS for Cu and Zn were 0.010, and 0.007 mg/L, respectively.

Before usage, all glassware and plastics were soaked overnight in 10% nitric acid, rinsed with distilled water, and dried. The goal is to keep any contamination to a minimum. Procedure blanks and triplicates of samples were also tested for quality control. Analytical blanks were also digested in the same way as the samples to check for contamination. To ensure the analytical quality, blanks were used in each series of assays at the same time. The digesting method for certified reference materials (CRM) was carried out in the same way. The CRM of dogfish liver was used to verify the method’s accuracy (DOLT-3, National Research Council Canada). The acquired results were consistent with verified values, demonstrating the method’s repeatability (Cu CRM = 31.2 mg/kg, Cu measurement = 32.9 mg/kg with CV = 1.16%; Zn CRM = 86.6 mg/kg, Zn measurement = 103 mg/kg with CV = 1.80%). The results of the recovery were satisfactory (106–119%).

### 2.4. Data Treatment for Human Health Risk Assessment

For human health risk assessment (HHRA), the two-metal data on a dry weight (dw) basis were converted into ww data by using a conversion factor of respective fish species as shown in Table S1. To estimate the HHRA derived from ingesting the fish, three assessments were made, namely:

(a) Direct comparisons with MPLs

In this study, three MPLs of Cu and Zn of seafood safety guidelines were used, namely those proposed by the FAO [67], the Ministry of Agriculture, Fisheries and Food [68], and Malaysian Food Regulation 1985 (MFR) [69].

(b) Estimation of THQ

To calculate the THQ, firstly, the estimated daily intake (EDI) was calculated. EDI is the estimation of the particular metal intake by using the body weight (BW) and fish consumption rate. It was calculated as in Equation (1):

EDI = (Mc × CR)/BW(1)

where,

Mc = Metal concentration in the fish muscles (mg/kg) on a ww basis.

CR = Fish consumption rate (100 g/person/day) for Malaysian adults based on 2675 respondents (Malay: 76.9%; Chinese: 14.7%; India: 8.4%) [70];

BW = Body weight employed was 62 kg for adult Malaysian population, according to Nurul Izzah et al. [70].

Later, the THQ was calculated in Equation (2):

THQ = EDI/ORD(2)

where,

ORD = Oral reference dose.

ORD is an estimate of the daily intake of a contaminant over a lifetime that would not be expected to cause adverse health effects [71]. The ORD values (Cu = 40; Zn = 300, µg/kg/day) provided by the USEPA regional screening level [72] were used in this study.

(c) Comparisons between estimated weekly intake (EWI) and provisional tolerable weekly intake (PTWI).

The Joint FAO/WHO Expert Committee on Food Additives (JECFA) set the PTWI [73]. By calculating weekly metal exposures and comparing the results to the prescribed PTWI values, the risk to human health from fish consumption was assessed. The PTWI is defined as the estimated quantity of a substance in food or drinking water that can be consumed weekly throughout a lifetime without posing a significant health risk, expressed in mg/kg BW [64].

As a result, calculations were conducted to figure out how many fish from this study exceeded the PTWI restrictions. According to JECFA [73,74], Cu and Zn have PTWIs of 3.50 mg/kg BW/week and 1.00 mg/kg BW/week, respectively, which were recalculated from the provisional maximum tolerable daily intake of 0.50 mg/kg BW/day, and 1.00 mg/kg BW/day, for Cu and Zn, respectively. As a result, for a 62 kg adult, the PTWIs are 217 mg/week for Cu and 434 mg/week for Zn. To estimate the risk of exposure from consuming fish, the estimated weekly intake (EWI) of the elements of fish was calculated as in Equation (3):

EWI= EDI × 7 (3)

where,

EDI = estimated daily intake calculated in Equation (1) and ×7, because of the 7 days in a week.

The comparison between calculated EWI and established PTWI limits for a 62 kg adult will determine whether the calculated EWI values are lower than the established PTWI of Cu and Zn.

## 3. Results and Discussion

Based on a total of 57 individuals of 19 species of fish from Setiu, the lengths ranged from 12.5 to 14.5 cm (*Carangoides malabaricus*) to 43.0–46.0 cm (*Trichiurus lepturus*), and the wet weights ranged from 30 to 45 g (*Selaroides leptolepis*) to 325–335 g (*Scomberomorus commerson*) (Table S1).

Out of the 19 species of marine fish and samples analyzed, the samples included 11 families, namely, Carangidae (six species), Sciaenidae (three species), Stromateidae (one species), Clupeidae (one species), Chirocentridae (one species), Scrombidae (two species), Dasyatidae (one species), Nemipteridae (one species), Lactariidae (one species), Trichiuridae (one species), and Ariidae (one species). Out of these families, they can be categorized into four major niche habitats, namely, reef-associated, benthopelagic, demersal, and pelagic-neritic (Table S1).

### 3.1. Comparison with Food Safety Guidelines of Cu and Reported Cu Concentrations in the Different Fish Species

**Figure 2:**
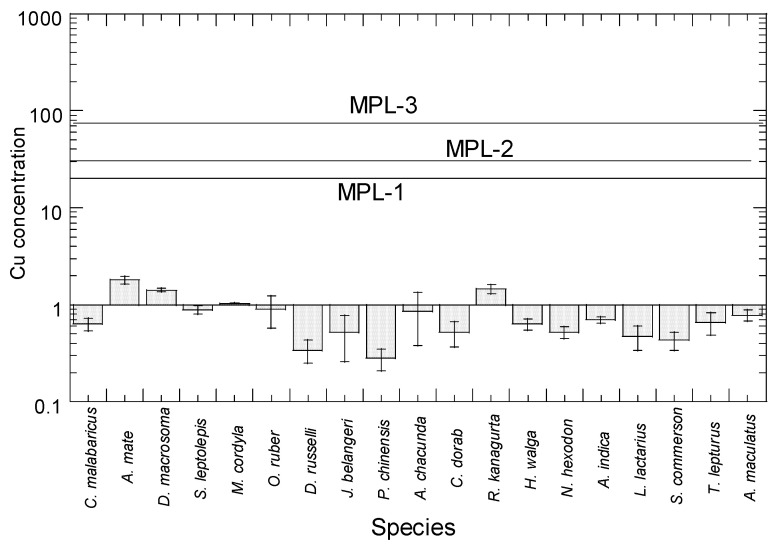
Total mean concentrations (mean ± SE, mg/kg wet weight) of Cu in 19 marine fish from Setiu, east coast of Peninsular Malaysia. Note: MPL-1 = MAFF [68]; MPL-2 = MFR [69]; MPL-3 = FAO [67]. Y-axis is based on logarithmic scale.

For Cu in the 19 species from Setiu, the concentrations ranged from 0.29 to 1.80 mg/kg ww (1.50–7.83 mg/kg dw) (Figure 2). The current Cu ranges (0.29–1.80 mg/kg ww) were well below the MPLs suggested by MFR (30 mg/kg ww) [69], MAFF (20 mg/kg ww) [68], and FAO [67], which suggested a range of 20–70 mg/kg ww for the legal limits of Cu. As a result, there was no evident Cu risk associated with eating Setiu fish.

Comparison of mean Cu concentrations between the present study and reported studies (15 species) of marine fish in literature are shown in Table S2. In the present study, the highest and lowest concentrations of Cu were found in *Atule mate* (1.80 mg/kg ww) and *Pampus chinensis* (0.29 mg/kg ww), respectively. The present Cu ranges (0.29–1.80 mg/kg ww) of 19 species of marine fish from Setiu (Table S2) were comparable to the Cu ranges (0.04–11.2 mg/kg ww) of 15 similar fish species from the present study with 82 reports of 38 papers (Table S2). Based on 46 species of marine fish collected from selected major fish landing ports of the Fisheries Development Authority of Malaysia and wholesale markets in Peninsular Malaysia, Wan Azmi et al. [58] reported that Cu from *M. cordyla* displayed the highest concentration of Cu (1.61 mg/kg ww) whereas *Otolithoides biauritus* displayed the lowest concentration of Cu (0.039 mg/kg ww).

Based on Figure 2, the Cu concentration was highest in *A. mate* (1.80 mg/kg ww), followed by *Decapterus macrosoma, Rastrelliger kanagurta, M. cordyla, Otolithes ruber,* and others. The Cu level (1.80 mg/kg ww) in *A. mate* was within the reported studies (0.92–1.53 mg/kg ww), where it was slightly higher than in Kuala Terengganu, and two times higher than concentrations found in the marine fish of Peninsular Malaysia (Table S2).

Cu level (1.42) in *D. macrosoma* was within the reported studies (0.70–11.2 mg/kg ww); it was also higher than concentrations found in Langkawi, the marine fish of Peninsular Malaysia, and the Gulf of Aqaba. However, concentrations were significantly lower (11.2 mg/kg ww) in Kuala Terengganu. For *R. kanagurta*, the concentration of Cu (1.45) from this study was within the range (0.28–3.30 mg/kg ww) reported in the literature (Table S2). The present Cu levels were higher than those collected from Mersing (east coast of Peninsular Malaysia), Indonesia and Thailand, Saint Martin Island, Andaman Sea, Peninsular Malaysia, Port Dickson, Pahang coastal waters, Cochin coast, Palk Bay, Kochi coast, and the west coast of Peninsular Malaysia. However, the Setiu’s Cu level was lower than concentrations found in Langkawi Island and the Kunduchi fish market in Dar es Salaam (Table S2). Rejomon et al. [75] and Nurnadia et al. [76] found Cu concentrations of 0.83 and 0.88 mg/kg ww in *R. kanagurta*, respectively.

For *M. cordyla,* the concentration of Cu (1.03 mg/kg ww) from this study was within the range (0.32–5.91 mg/kg ww) reported in the literature (Table S2). The Setiu Cu level was higher than concentrations found in Langkawi, Port Dickson, Kelantan, Cambodia, Thailand, Kuala Kedah, Port Dickson, Pahang coastal waters, Mersing, and Cochin coast. However, it was lower than concentrations found in Tanjung Sepat (Selangor), the marine fish of Peninsular Malaysia, and the west coast of Peninsular Malaysia. Meanwhile, Nurnadia et al. [76] found 1.56 mg/kg ww of Cu in *M. cordyla*, which was higher than the concentration of similar species found in the present study (1.03 mg/kg ww).

For *O. ruber,* the concentration of Cu (0.90 mg/kg ww) from this study was within the range (0.19–2.98) reported in the literature. The Setiu Cu level was higher than concentrations found in Khlong Yai, Chabahar Bay, the marine fish of Peninsular Malaysia, the Kuala Tanjung coast (North Sumatra), and the Northwest coastal Karachi (Pakistan). However, it was lower than concentrations found in Miri, North of the Persian Gulf, the Khuzestan shore (northwest of the Persian Gulf), and Kharg Island (Persian Gulf) (Table S2). For *S. leptolepis*, the concentration of Cu (0.89 mg/kg ww) from this study was within the range (0.50–1.41 mg/kg ww) reported in the literature (Table S2). The Setiu Cu level was higher than concentrations found in the marine fish of Peninsular Malaysia, Port Dickson, and Pahang coastal waters but lower than concentrations found on the west coast of Peninsular Malaysia. In addition, the concentration Cu in *S. leptolepis* from this study (0.90 mg/kg ww) is also lower than in another study (1.41 mg/kg ww) by Nurnadia et al. [76].

For *Johnius belangeri*, the concentration of Cu (0.52 mg/kg ww) from this study was within the range (0.11–0.69 mg/kg ww) reported in the literature (Table S2). The Setiu Cu level was higher than concentrations found in Kapar, Mersing, the Kuala Tanjung coast (North Sumatra), and the Musa estuary (Iran) but lower than the Blanakan river estuary (Indonesia). For *P. chinensis*, The Setiu Cu level (0.29 mg/kg ww) was within the range (0.04–3.08 mg/kg ww) of reported studies; it was higher than concentrations found in the Karachi fish harbor (Pakistan) and Cox’s Bazar (Bangladesh), but lower than concentrations found in northwest coastal Karachi (Pakistan), the Karnaphuli River estuary, (Bangladesh), and southeastern Bangladesh (Table S2).

For *Anodontostama chacunda*, the concentration of Cu (0.86 mg/kg ww) from this study was within the range (0.34–1.83 mg/kg ww) reported in the literature (Table S2). It was higher than concentrations found in Lada Bay (Indonesia) and Bondet (Indonesia), but lower than concentrations found in Kuala Terengganu and the Arabian Sea coast (Pakistan). For *Chirocentrus dorab*, the concentration of Cu (0.52 mg/kg ww) from this study was within the range (0.35–1.14 mg/kg ww) reported in the literature (Table S2). It was higher than concentrations found on the Cochin coast (India) but lower than concentrations found in Palk Bay (India) and the west coast of Peninsular Malaysia, and was relatively lower than in another study (0.99 mg/kg ww) by Nurnadia et al. [76].

For *S. commerson*, the concentration of Cu (0.43 mg/kg ww) from this study was within the range (0.30–2.90 mg/kg ww) reported in the literature (Table S2). It was higher than concentrations found in Koh Kong (Cambodia), the marine fish of Peninsular Malaysia and Zhongsha (South China Sea) but lower than concentrations found on Langkawi Island and the coast of Karachi (Pakistan). For *T. lepturus*, the concentration of Cu (0.66 mg/kg ww mg/kg ww) from this study was within the range (0.46–1.98 mg/kg ww) reported in the literature. It was higher than concentrations found on Kutubdia Island and in the Mumbai harbor (India), but lower than concentrations found in Miri (Table S2).

For *C. malabaricus*, the concentration of Cu (0.63 mg/kg ww) from this study was higher than the only report (0.09 mg/kg ww) from the Andaman Sea. For *Dendrophysa russelli,* the concentration of Cu (0.35 mg/kg ww) from this study was lower than the only report (0.38 mg/kg ww) from the Mumbai harbor, India (Table S2). For *Arius maculatus,* the concentration of Cu (0.78 mg/kg ww) from this study was higher than the only report (0.40 mg/kg ww) from the Mumbai harbor, India (Table S2).

The Cu ranges (0.29–1.80 mg/kg ww) found in this investigation are mostly consistent and slightly higher than those found in the literature. For example, based on 49 commercial fish species from the eastern Mediterranean Sea (Izmir Outer Bay, Homa Lagoon/Izmir, and Mersin Bay), Celik and Oehlenschläger [77] reported Cu levels ranging from 0.12 to 1.14 mg/kg ww. Simanjuntak et al. [78] reported Cu ranges of 0.09–0.35 mg/kg ww based on eight marine fish of North Sumatra. Türkmen et al. [45] reported levels of Cu (0.51–7.05 mg/kg ww) in muscles of twelve fish species from the Aegean Sea and the Mediterranean Sea. Based on ten different fish species from the Black Sea, Tuzen [79] reported the levels as 0.65–2.78 mg/kg ww for Cu. Based on fourteen benthic and pelagic fish species collected from three main landing areas (Shalateen, Hurghada, and Suez) in the Egyptian Red Sea, El-Moselhy et al. [19] reported a Cu range from 0.17 to 0.77 mg/g ww. Based on the muscle of 17 species over a five-year period in several surface water systems in eastern Tennessee, Blevins and Pancorbo [80] reported mean Cu levels in the muscle of different species of fish from nine stations ranging from 0.12 to 2.20 mg/kg ww.

The present Cu ranges (1.50–7.83 mg/kg dw) of 19 species from Setiu were comparable to Ong et al. [16]’ s findings that reported five marine species from Setiu, with Cu levels ranging from 0.69 to 3.04 mg/kg dw. The highest concentrations of Cu were: *M. cordyla* (3.04 mg/kg dw), followed by *Selaroides* sp. (1.36 mg/kg dw), *Rastrelliger* sp. (1.29 mg/kg dw). Cu concentrations in fish muscle were reported to range from 0.86 to 3.48 mg/kg dw in Malaysian coastal waters [54].

The present Cu ranges were also lower than those reported in the literature. Based on seven marine fish species collected from the Miri coast, Anandkumar et al. [81] reported that the Cu concentration in fish muscles varied between 8.50 and 13.3 mg/kg dw, comparable to the Cu concentration of the marine fish from the Mediterranean Sea (3.40–5.88 mg/kg dw) [82], the Turkish Sea, (0.16–10.7 mg/kg dw) [83], and the Palk Bay of India (0.90–8.68 mg/kg dw) [84] but lower than the values reported from Poompuhar, SE coast of India (20.5 mg/kg dw) [85] and Langkawi Island, Malaysia (11.5–13.9 mg/kg dw) [57]. Collected from the Mumbai harbor, the Cu values in fish muscle samples ranged from 0.87 to 6.51 mg/kg dw, according to Velusamy et al. [27]. Cu concentrations were found in the highest concentration in *A. arius* (6.51 mg/kg dw) and the lowest in *J. macropterus* (0.87 mg/kg dw).

Cu levels in the muscle tissues of fish species collected off Mangalore and off Kochi ranged from 2.06 to 3.09 mg/kg dw and 2.66 to 3.62 mg/kg dw, respectively, with a maximum for *Caranx melampygus* and a minimum for *Lates calcarifer*, *Nemipterus japonicus*, *R. kanagurta*, and *Cyanoglossus macrostomus*, according to Rejomon et al. [75]. Based on fish from the Meghna river estuary (Bangladesh), Jahangir Sarker et al. [38] reported the Cu concentrations as 4.63–73.6 mg/kg dw. Cu concentrations were reported to be 0.73–1.83 mg/kg dw in nine fish species from Turkey’s Black and Aegean Seas [46]. Dural Eken et al. [86] reported the Cu concentrations in the three marine fish species caught in Turkey’s Tuzla Lagoon ranged from 0.26 to 0.82 mg/kg dw. In bluefin tuna caught in the northwest Atlantic off Newfoundland, Hellou et al. [87] reported the mean Cu concentration in muscle tissue as 1.00 mg/kg dw. In cod caught off the coast of Newfoundland, mean Cur concentrations of <1.2–1.5 mg/kg dw in fish muscle were found [87].

### 3.2. Health Risk Assessment of Cu

Values of EDI, THQ, and EWI of Cu calculated based on the present study and citing Cu data in the marine fish from the literature are shown in Table S3. Overall statistics of Cu concentrations (mg/kg ww), EDI, THQ, and EWI in the 19 marine fish species of marine fish from Setiu are presented in Table 1. Overall statistics of Cu concentrations (mg/kg ww) with recalculation of EDI, THQ, and EWI in the 15 marine fish species cited from the literature (82 reports of 38 papers) are given in Table 2.

The Cu EDI values ranged from 0.46 to 2.90 while Cu THQ values ranged from 0.01 to 0.07 (Table S3). The THQ values of Cu were lower than one in all 19 fish species, indicating low non-carcinogenic risk and considered safe for human consumption. This also demonstrates the absence of public health hazards in Cu risk. Consumption of contaminated fish, on the other hand, may result in an increase in Cu content in people. In comparison to the worldwide guideline, this study found that the Cu concentration is within an acceptable range. Similarly, Yabanli and Alparslan [88] also reported lower EDI values. The EDI value for adults and children aged 12 years are 0.07 and 0.04, respectively, for Cu. Yabanli and Alparslan [88] also reported lower THQ for adults and children. The THQ for adults and children was also below 1.00. A study by Praveena and Lin [89], revealed that the THQ values calculated for Cu in marine fish collected from Port Dickson (west coast of Peninsular Malaysia) were also less than one, indicating that there were no Cu adverse effects via fish consumption.

From Table 1, the estimated Cu EWI ranged between 3.21 and 20.3 µg/kg BW/week, with *P. chinensis* being the lowest and *A. mate* being the highest. The computed EWI was found to be lower than the Cu (3500 (µg/kg BW/week) determined PTWI. According to FAO/WHO JECFA guidelines, intake of the examined fish does not represent a risk of Cu poisoning to humans. Based on nine heavy metals in 46 species of marine fish from the coastal waters of Peninsular Malaysia, Wan Azmi et al. [58] reported that the estimated Cu EWI ranged from 0.69 and 27.65 (µg/kg BW/week). Based on 82 reported data of Cu in marine fish, the Cu EWI ranged from 0.43 to 126 (µg/kg BW/week) with *D. macrosoma* collected from Kuala Terengganu [15], as the highest. The EWI of Cu was reported to be between 0.480 and 1.279 g/kg BW/week in a study by Peycheva et al. [90] in Bulgaria, which was around 25% lower than our findings.

Türkmen et al. [45] reported the EDI and EWI values of Cu for economically important fish species consumed by adult people in Turkey. These values were estimated by assuming that a 70-kg person will consume 20 g fish/day, which is equal to 140 g fish/week. When compared, the estimated EWI values ranged from 165 to 987 µg/70 kg BW/week for eight species of economically important fish collected from Aegean and Mediterranean seas and were far below the recommended values PTWI of 3500 µg/kg BW/week and PTWI of 245,000 µg/70 kg BW/week for a 70 kg adult person [73].

The total daily intake of Cu in adults varies between 0.9 and 2.2 mg/day. Intake in children has been estimated to be 0.6–0.8 mg/day (0.07–0.1 mg/kg BW/day) [4]. The lower limit of the acceptable range of oral intake is 20 µg Cu/kg BW/day. In infancy, it is 50µg Cu/kg BW/day [4]. The estimated daily intake of Cu from food is 1.0–1.3 mg/day for adults (0.014–0.019 mg/kg/day) [2]. In the United States, the median intake of Cu from food is 0.93–1.3 mg/day for adults (0.013–0.019 mg Cu/kg BW/day using a 70-kg reference of BW) [2].

### 3.3. Comparison with Food Safety Guidelines of Zn and Reported Zn Concentrations in the Different Fish Species

**Figure 3:**
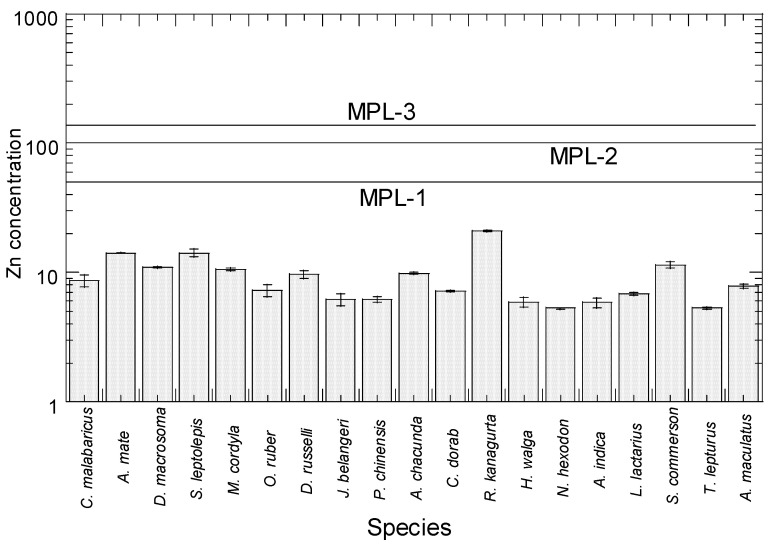
Total mean concentrations (mean ± SE, mg/kg wet weight) of Zn in 19 marine fishes from Setiu, east coast of Peninsular Malaysia. Note: MPL-1 = MAFF [68]; MPL-2 = MFR [69]; MPL-3 = FAO [67]. Y-axis is based on logarithmic scale.

For Zn in the 19 species from Setiu, the concentrations ranged from 5.29 to 20.9 mg/kg ww (24.1–80.5 mg/kg dw) (Figure 3). The present Zn ranges were below the MPLs (40–150 mg/kg ww) suggested by FAO [67], the MAFF (50 mg/kg ww) [68], and the MFR [69] (100 mg/kg ww). As a result, there was no evident Zn risk associated with eating Setiu fish.

Comparisons of mean Zn concentrations (mg/kg dw and ww) in various species (15 species) of marine fish reported in the literature are given in Table S4. The comparison of mean Zn concentrations between the present study and the reported studies (15 species) of marine fish in literature is shown in Table S4. The highest and lowest concentrations of Zn were found in *R. kanagurta* (20.93 mg/kg ww) and *Nemipterus hexodon* (5.29 mg/kg ww), respectively. According to Ahmed et al. [14], the mean Zn concentrations ranged from 5.19 to 20.6 mg/kg ww, with *R. kanagurta* having the highest Zn concentration (20.6 mg/kg ww), and *S. leptolepis* (14.3 mg/kg ww) and *A. mate* having the lowest Zn content (12.3 mg/kg ww).

The Cu concentration was highest in *R. kanagurta* (20.93 mg/kg ww), followed by *A. mate*, *S. leptolepis*, *D. macrosoma*, *S. commerson*, and others (Figure 3). For *R. kanagurta*, the Setiu Zn level (20.93 mg/kg ww) was higher than all the reported studies from the literature (2.39–13.18 mg/kg ww), except for the concentrations found in the Kunduchi fish market in Dar es Salaam (Tanzania) (27.0 mg/kg ww). The concentrations lower than the present study included those found in Mersing, Indonesia, Thailand, Bangladesh, Andaman Sea, the marine fish of Peninsular Malaysia, Pahang coastal waters, Cochin coast (India), Palk Bay (India), Langkawi Island, west coast of Peninsular Malaysia, and coastal waters off Kochi (India) (Table S4).

For *A. mate,* the present Zn level (14.1 mg/kg ww) was higher than concentrations found in Kuala Terengganu (6.72 mg/kg ww) and the marine fish of Peninsular Malaysia (8.49 mg/kg ww). For *S. leptolepis,* the Zn level (14.11 mg/kg ww) was higher than all the reports (2.64- 7.20 mg/kg ww) in the literature, namely, in the marine fish of Peninsular Malaysia, Pahang coastal waters, and the west coast of Peninsular Malaysia. For *D. macrosoma*, the present Zn level (10.97 mg/kg ww) was within the ranges (4.06–15.90 mg/kg ww) reported in the literature, namely, it was higher than concentrations found in Langkawi, Kuala Terengganu, and the Gulf of Aqaba (Jordan), but lower than those found in the marine fish of Peninsular Malaysia. *Decapterus macrosoma* had the greatest Zn (63.01 mg/kg dw) concentration, according to Ong et al. [16]. The levels were, however, lower than those found in a study by Khalaf et al. [20] (Zn 94.57 mg/kg dw). In *D. macrosoma*, Agusa et al. [54] found substantially lower levels of Zn (29.1 mg/kg dw). According to Wan Azmi et al. [58], Zn had the highest level of accumulation, with *D. macrosoma* having the highest concentration (15.9 mg/kg) (Table S4).

For *S. commerson,* the concentration of Zn (11.41 mg/kg ww) from this study was higher than all the reported studies from the literature (2.17–8.56 mg/kg ww), namely from Koh Kong (Cambodia), the marine fish of Peninsular Malaysia, Langkawi Island, the coast of Karachi (Pakistan), and Zhongsha (South China Sea). For *M. cordyla*, the Zn level (10.52 mg/kg ww) was higher than all the reports (2.30–8.10 mg/kg ww) in the literature, namely from Langkawi, Port Dickson, Kelantan, Cambodia, Thailand, Marine fish Peninsular Malaysia, Pahang coastal waters, Mersing, the Karachi fish harbor (Pakistan), the Cochin coast (India), and the west coast of Peninsular Malaysia. Collected from Setiu, Ong et al. [16] reported Zn concentrations in *M. cordyla* as 8.97 mg/kg dw (Table S4).

For *O. ruber,* the Zn level (7.26 mg/kg ww) was within the Zn ranges of all the reports (1.13–7.69 mg/kg ww) in the literature. It was higher than those from Khlong Yai (Thailand), Miri, Chabahar Bay (Iran), the marine fish of Peninsular Malaysia, the northern part of the Hormuz strait (Persian Gulf), the southwest coast of Peninsular Malaysia, and Kharg Island (the Persian Gulf, but lower than the Kuala Tanjung coast (Indonesia). For *J. belangeri,* the Zn level (6.16 mg/kg ww) was within the Zn ranges of all the reports (3.02–7.23 mg/kg ww) in the literature. The present Zn level was higher than those in Kapar, Mersing, and Daya Bay’s Fishery Resource Reserve (South China Sea), but lower than those in the Blanakan river Estuary (Indonesia), and the Kuala Tanjung coast (North Sumatra) (Table S4).

For *P. chinensis*, the Zn level (6.19 mg/kg ww) was within the Zn ranges of all the reports (1.52–13.05 mg/kg ww) in the literature. The present Zn level was higher than those found in the Karachi fish harbor (Pakistan), Kalimati fish market (Kathmandu), and southeastern Bangladesh but lower than those found in Cox’s Bazar (Bangladesh) (13.05 mg/kg ww). For *A. chacunda,* the Zn level (9.81 mg/kg ww) was within the Zn ranges of all the reports (1.15–12.9 mg/kg ww) in the literature. The present Zn level was higher than those found in Lada Bay (Indonesia), Bondet (Indonesia), and Kuala Terengganu, but lower than those found in the Arabian Sea coasts of Pakistan (12.9 mg/kg ww) (Table S4).

For *C. dorab*, the Zn level (7.14 mg/kg ww) was higher than all reports (0.76–6.85 mg/kg ww) in the literature, namely from Cochin coast (India), Palk Bay (India), the southwest coast of Peninsular Malaysia, and the west coast of Peninsular Malaysia. Nurnadia et al. [76] reported Zn concentration (3.40 mg/kg ww) in *C. dorab*.

For *T. lepturus*, the concentration of Zn (5.31 mg/kg ww) from this study was lower than all the reported studies from Miri, Kutubdia Island, and the Mumbai harbor (India) in the literature. For *C. malabaricus,* the present Zn level (8.63 mg/kg ww) was lower than the only report found from the Andaman Sea (4.74 mg/kg ww). For *Dendrophysa russelli,* the present Zn level (9.65 mg/kg ww) was lower than the only report (8.90 mg/kg ww) from the Mumbai harbor (India). For *A. maculatus,* the present Zn level (7.82 mg/kg ww) was lower than the only report (12.7 mg/kg ww) from the Mumbai harbor (India) (Table S4).

The present Zn ranges (5.29–20.93 mg/kg ww) of 19 species of marine fishes from Setiu (Table S5) were comparable to and within the Zn ranges (0.76–27.04 mg/kg ww) of 15 similar fish species from the present study with 76 reports of 35 papers (Table S5). Based on 46 species of marine fish collected from selected major fish landing ports of the Fisheries Development Authority of Malaysia and wholesale markets in Peninsular Malaysia, Wan Azmi et al. [58] reported the median ranges of Cu were 2.30–15.9 mg/kg ww, with the highest Zn level found in *D. macrosoma* (15.9 mg/kg ww), whereas *Otolithoides biauritus* displayed the lowest concentration of Zn (2.30 mg/kg ww).

Babji et al. [50] reported Zn levels of six species of fishes caught at six different locations from Peninsular Malaysia ranged from 2.30 to 6.50 mg/kg ww. However, none of the six species were among the 19 species from the present study. Simanjuntak et al. [78] reported Zn ranges ranging from 2.97 to 11.5 mg/kg ww of fish collected from North Sumatra. Based on 49 commercial fish species from the eastern Mediterranean Sea (Izmir Outer Bay, Homa Lagoon/Izmir, and Mersin Bay), Celik and Oehlenschläger [77] reported Zn levels ranging from 2.38 to 9.73 mg/kg ww. Türkmen et al. [45] reported levels of Zn (3.51–53.5 mg/kg ww) in muscles of twelve fish species from the Aegean Sea and the Mediterranean Sea. Based on ten different fish species from the Black Sea, Tuzen [79] reported the levels as 38.8–93.4 mg/kg ww for Zn.

Arulkumar et al. [84] reported that the highest concentration of Zn was observed in *P*. *pelagicus* (55.1 mg/kg ww), followed by *S*. *brevimana* (52.1 mg/kg ww) and *S*. *aculeate* (42.8 mg/kg ww). Based on fourteen benthic and pelagic fish species collected from three main landing areas (Shalateen, Hurghada, and Suez) in the Egyptian Red Sea, El-Moselhy et al. [19] reported Zn ranges from 1.17 to 12.0 mg/kg ww. In the Toronto harbor, Ontario, Canada, various species of fish contain only slightly elevated Zn levels (36.0 mg/kg ww) in muscle tissues [5].

The present Zn ranges (24.1–80.5 mg/kg dw) of 19 species from Setiu were comparable and lower than those reported in the literature. Collected from the Setiu loading site, Ong et al. [16] reported five marine species from Setiu with Zn ranging from 5.81 to 11.2 mg/kg dw. The highest concentrations of Cu were: *M. cordyla* (8.97 mg/kg dw), followed by *Selaroides* sp. (10.4 mg/kg dw) and *Rastrelliger* sp. (8.42 mg/kg dw). Bashir et al. [91] found that Zn concentrations ranged from 30.2 to 13.1 mg/kg dw in *Arius thalassinus* and *J. belangeri*. In a study conducted on the eastern coast of Malaysia, Fathi et al. [51] discovered that *M. cordyla* had the lowest mean Zn concentration (17.5 mg/kg dw), while Kamaruzzaman et al. [61] found Zn levels ranging from 12.0 mg/kg dw (*S. leptolepis*) to 25.0 mg/kg dw (*R. kanagurta*), collected from Pahang coastal waters.

Based on seven marine fish species collected from the Miri coast, Anandkumar et al. [81] reported that the Zn concentration in fish muscles varied between 16.9 and 71.0 mg/kg dw. In the muscles of Malaysian marine fish, Zn concentrations ranged from 15.4 to 60.1 mg/kg dw [54]. According to a study conducted on the Malaysian island of Langkawi, all fish species exhibited higher Zn concentrations than other metals, with Zn concentrations in muscles ranging from 34.3 to 49.4 mg/kg dw [57]. Irwandi and Farida [57] reported that the Zn levels of the fish caught in Pulau Tuba ranged from 34.3 (*R. kanagurta*) to 49.4 mg/kg dw (*Lutjanus johnii*). Rejomon et al. [75] reported that the Zn levels in the muscle tissue of different species of fish show wide fluctuations and range from 24.4 to 79.3 mg/kg dw and 37.4 to 84.3 mg/kg dw for the fish collected off Mangalore and off Kochi, respectively, with a maximum concentration observed for *Lates calcarifer* and a minimum for *Rasterelliger kanagurta* and *Cyanoglossus macrostomus*.

Bashir et al. [91] found Zn concentrations of 18.3 and 20.5 mg/kg dw in the muscles of *J. belangeri* and *A. thalassinus*, respectively, in fish samples from Kapar. Meanwhile, the Zn concentrations in the muscles of *J. belangeri* and *A. thalassinus* in Mersing fish samples were 13.1 mg/kg and 30.2 mg/kg dw, respectively. Kalay et al. [82] reported Zn ranges (14.1–33.5 mg/kg dw) in fish species caught from the Mediterranean Sea. Based on fish from the Meghna river estuary (Bangladesh), Jahangir Sarker et al. [38] reported Zn concentrations of 39.5–180 mg/kg dw. Uluozlu et al. [46] reported Zn ranges in fish from the Black and Aegean Seas, with Zn values ranging from 35.4 to 106 mg/kg dw. Dural Eken et al. [86] reported Zn concentrations in the three marine fish species caught in Turkey’s Tuzla Lagoon ranging from 8.27 to 75.4 mg/kg dw. The mean Zn concentration in muscle tissue of tuna (*Thunnus thynnus*) collected from the northwest Atlantic Ocean ranged from 12.0 to 25.0 mg/kg dw in 1990 [87].

### 3.4. Health Risk Assessment of Zn

The values of EDI, THQ, and EWI calculated based on the present study and citing Zn data in the marine fish from the literature are presented in Table S5. The overall statistics of Zn concentrations (mg/kg ww), EDI, THQ, and EWI in the 19 marine fish species of marine fishes from Setiu (N = 19) are presented in Table 3. The overall statistics of Zn concentrations (mg/kg ww) with recalculation of EDI, THQ, and EWI in the 15 marine fish species cited from the literature (76 reports of 35 papers) are presented in Table 4.

The Zn EDI values ranged from 8.53 to 33.8 while the Zn THQ values ranged from 0.03 to 0.11 (Table 4). The THQ values of Zn were lower than one in all 19 fish species, indicating a low non-carcinogenic risk of Zn and considered safe for human consumption. This also demonstrates an absence of public health hazards in Zn risk.

From Table 3 and Table 4, the estimated Zn EWI values ranged from 59.7 to 236 µg/kg BW/week, with *Nemipterus hexodon* being the lowest and *R. kanagurta* being the highest. The results demonstrated that all the values of calculated EWI were well below the established PTWI of Zn (7000 µg/kg BW/week). Therefore, the consumption of the studied fish would not pose adverse effects of Zn to consumers based on FAO/WHO JECFA guidelines. Based on nine heavy metals in 46 species of marine fish from the coastal waters of Peninsular Malaysia, Wan Azmi et al. [58] reported that the estimated Zn EWI ranged from 41.6 to 288 (µg/kg BW/week). Based on 73 reported data of Zn in marine fish, the Zn EWI ranged from 12.7 to 305 (µg/kg BW/week) with *R. kanagurta* collected from the Kunduchi fish market in Dar es Salaam, Tanzania [39], as the highest. Wan Azmi et al. [58] reported the EWI of Zn in the range of 7.3–15.9 µg/kg BW/week. The calculated HQ also demonstrated that HQ values were lower than 1, which implied that fish consumption from Peninsular Malaysia has a low non-cancer risk towards humans. The study by Peycheva et al. [90] also demonstrated that their HQ values for Zn (0.0005 to 0.0010) were lower compared to our study.

Turkmen et al. [45] reported the EDI and EWI values of Zn for economically important fish species consumed by adult people in Turkey. These values were estimated by assuming that a 70-kg BW person will consume 20 g fish/day, which is equal to 140 g fish/week. When compared, the estimated EWI values ranged from 532 to 7490 µg/70 kg BW/week for eight species of economically important fish collected from Aegean and Mediterranean seas, and were far below the recommended values PTWI (7000 µg/kg BW/week) [73], except for *Serranus scriba* (7490 µg/70 kg BW/week). However, all Zn EWI values were far lower than the PTWI of 490,000 µg/kg BW/week for the 70 kg adult person [73].

Estimated ranges of daily dietary intakes of total Zn are 5.6−10 mg/day for infants and children aged 2 months–11 years, 12.3–13.0 mg/day for children aged 12–19 years, and 8.8−14.4 mg/day for adults aged 20–50 years [5]. The average daily Zn intake in the diet in this country ranges from 5.2 to 16.2 milligrams (milligram = 0.001 g). The National Academy of Sciences estimated an RDA for Zn of 11 mg/day (men). Eleven mg/day is the same as 0.16 mg/kg BW/day for an average adult male (70 kg). An RDA of 8 mg/day, or 0.13 mg/kg of BW for an average adult female (60 kg), was established for women because they usually weigh less than men. Lower Zn intake was recommended for infants (2–3 mg/day) and children (5–9 mg/day) because of their lower average BWs [3].

### 3.5. Higher Metals in the Pelagic Than Demersal Fishes

From Table 5, it is demonstrated that the mean concentrations of Cu followed the order of reef-associated > pelagic-neritic > benthopelagic > demersal. For Zn, they followed the order of pelagic-neritic > reef-associated > demersal > benthopelagic. Wan Azmi et al. [58] also reported that the median concentrations (mg/kg ww) of Cu and Zn in pelagic fish (Cu: 0.91; Zn: 7.17) were significantly (*p* < 0.05) higher compared to demersal fish (Cu: 0.27; Zn: 4.02). This was clearly indicated in the Cu and Zn levels from the present study. However, the mean Zn level in the demersal fish was slightly higher than the benthopelagic fish.

However, the results of the present study and Wan Azmi et al. [58] based on fish caught from Peninsular Malaysian coasts disagreed with the predicted lower levels of metals in the bodies of pelagic fishes when compared to those in demersal fishes, as indicated by several studies [27,92]. Because the demersal fishes interact directly with sediments and spend most of their lives on top of silt and soil, absorbing more metals from the bottom of the sea, they frequently have a high concentration of metals in their bodies [19,92]. Thus, the risk of contamination is predicted to be higher in demersal than in pelagic organisms [93].

The higher levels of Cu and Zn in the pelagic rather than demersal fish could be due to land-based discharges and surface run-off from Peninsular Malaysia, which could have brought higher nutrients and chemicals to the surface waters that have become a major niche habitat for reef-associated, pelagic-neritic, and benthopelagic fishes.

It should also be noted that the efficiency of the uptake of PTMs from contaminated water and food varies based on ecological needs, body metabolic capability, and the environmental parameters of salinity and temperature [94]. Metal accumulated in many fish species could vary depending on habitats and ecological requirements, metabolic capabilities, and eating habits [95,96].

### 3.6. Relationships of Metal Concentrations and Body Size of Fish

**Figure 4:**
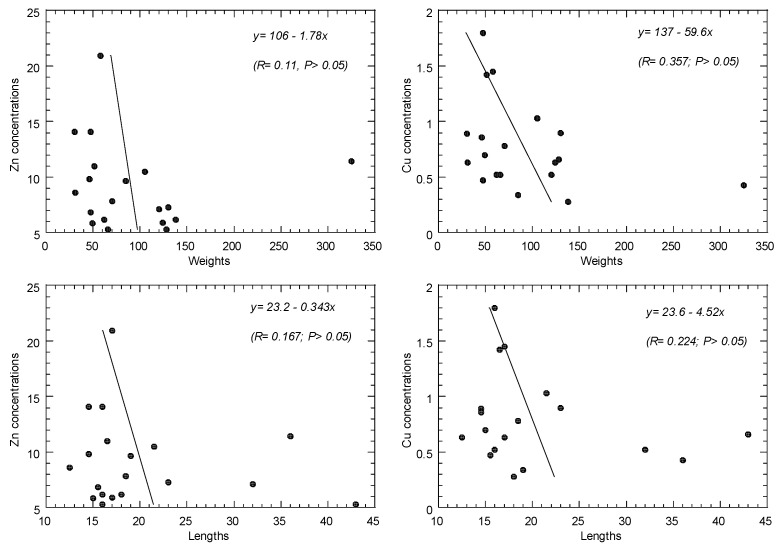
Relationships between metal concentrations (mg/kg wet weight) and body lengths (g) (and body weight (g)) in the 19 species of fish from Setiu, Terengganu.

The relationships between metal concentrations, body lengths, and body weight in the 19 species of fish from Setiu are presented in Figure 4. Overall, there are insignificant differences (*p* > 0.05) between the levels of Cu and Zn and body size (length and weight). Knowledge from the literature demonstrates that such relationships are inconsistent.

Wan Azmi et al. [49] found no significant difference (*p* > 0.05) between Cu (and Zn) and fish body length in 46 species of marine fish from the coastal waters of Peninsular Malaysia. Significant positive significant (*p <* 0.05) relationships between total fish length and weight and heavy metal concentrations were also reported by Bashir et al. [81].

Using linear regression analysis, Yi and Zhang [97] investigated the correlations between fish size (length and weight) and metal concentrations in seven fish species obtained from the Yangtze River (China). In most cases, they found positive associations between fish sizes and metal levels, with the exception of mercury and chromium levels in the sizes of catfish and yellow-head catfish, which showed negative relationships. Canli and Atli [98] looked at the association between fish size (length and weight) and metal concentrations in the tissues of six fish species obtained from the Mediterranean Sea’s northeast. They concluded that, with the exception of a few cases, there was no substantial relationship between metal concentrations and fish size.

## 4. Conclusions

In conclusion, the detected concentrations of Cu and Zn in the samples collected from Setiu were below the MPLs, and THQs for all species were below one. This indicates no non-carcinogenic risks of Cu and Zn for consumers. It was also found that the calculated values of EWI were lower than the PTWI of Cu and Zn. Even though the EWI of the population was lower than the PTWI levels, the excessive consumption of fish could lead to adverse effects on human health.

Various PTMs accumulated at different rates in different species, according to the findings. To further understand the processes affecting the accumulation of PTMs in fish species, more research on physiological and ecological factors is proposed. In the future, a continuous monitoring program using a validated questionnaire should be implemented to acquire data on the actual consumption rates of each fish species among local populations (adult male and female/pregnant women, and children with different age groups). This information is crucial in determining the actual chances of developing non-carcinogenic/chronic systemic effects after consuming each species. Finally, it is suggested that PTM contamination of commercial marine fish species be monitored regularly to ensure the safety of fish consumption. 

## Figures and Tables

**Table 1:** Overall statistics of Cu concentrations (mg/kg wet weight), estimated daily intake (EDI), target hazard quotient (THQ), and estimated weekly intake (EWI) in the 19 marine fish species of marine fish from Setiu (N = 19).

	WW	EDI	THQ	EWI
Minimum	0.29	0.46	0.0115	3.22
Maximum	1.80	2.90	0.0726	20.33
Sum	14.82	23.90	0.5975	167.31
Mean	0.78	1.26	0.0314	8.81
Median	0.66	1.06	0.0265	7.43
SD	0.40	0.65	0.0163	4.55
SE	0.09	0.15	0.0037	1.04

Note: SD = standard deviation; SE = standard error.

**Table 2:** Overall statistics of Cu concentrations (mg/kg wet weight) (WW) with recalculation of estimated daily intake (EDI), target hazard quotient (THQ), and estimated weekly intake (EWI) in the 15 marine fish species cited from the literature (82 reports of 38 papers) (N = 82).

	WW	EDI	THQ	EWI
Minimum	0.04	0.06	0.0015	0.43
Maximum	11.20	18.06	0.4516	126.45
Mean	1.04	1.68	0.0421	11.79
Median	0.64	1.03	0.0256	7.18
SD	1.45	2.34	0.0586	16.40
SE	0.16	0.26	0.0065	1.81

Note: SD = standard deviation; SE = standard error.

**Table 3:** Overall statistics of Zn concentrations (mg/kg wet weight), estimated daily intake (EDI), target hazard quotient (THQ), and estimated weekly intake (EWI) in the 19 marine fish species of marine fish from Setiu (N = 19).

	WW	EDI	THQ	EWI
Minimum	5.29	8.53	0.0280	59.70
Maximum	20.93	33.75	0.1130	236.30
Mean	9.15	14.76	0.0492	103.32
Median	7.82	12.61	0.0420	88.30
SD	3.96	6.38	0.0214	44.71
SE	0.91	1.46	0.0049	10.26

Note: SD = standard deviation; SE = standard error.

**Table 4:** Overall statistics of Zn concentrations (mg/kg wet weight) (WW) with recalculation of estimated daily intake (EDI), target hazard quotient (THQ), and estimated weekly intake (EWI) in the 15 marine fish species cited from the literature (76 reports of 35 papers) (N = 76).

	WW	EDI	THQ	EWI
Minimum	0.76	1.23	0.0040	8.60
Maximum	27.04	43.61	0.1450	305.30
Mean	6.08	9.81	0.0327	68.66
Median	5.53	8.92	0.0295	62.35
SD	3.86	6.23	0.0207	43.63
SE	0.44	0.72	0.0024	5.01

Note: SD = standard deviation; SE = standard error.

**Table 5:** Comparison of concentrations (minimum-maximum (mean), mg/kg wet weight) of Cu and Zn in the fish of different habitats from the present study.

Metal	Habitat			
Cu	Reef-associated (N = 7)	Pelagic-neritic (N = 4)	Benthopelagic (N = 3)	Demersal (N = 5)
	0.52–1.80 (1.00)	0.42–1.42 (0.79)	0.29–0.90 (0.61)	0.34–0.79 (0.56)
Zn	Pelagic-neritic (N = 4)	Reef-associated (N = 7)	Demersal (N = 5)	Benthopelagic (N = 3)
	6.86–20.55 (12.1)	5.80–14.26 (10.25)	5.19–9.53 (6.97)	5.25–7.27 (6.26)

## Data Availability

Not applicable.

## Supplementary Materials

The following supporting information can be downloaded at: https://www.mdpi.com/2305-6304/10/2/52/s1, Table S1: Description of the studied marine fishes, biometric features, water contents, and conversion factors; Table S2: Comparisons of mean Cu concentrations (mg/kg dry weight (DW) and wet weight (WW)) between the present study and reported studies (15 species) of marine fishes in literature; Table S3: Values of estimated daily intake (EDI), target hazard quotient (THQ), and estimated weekly intake (EWI) of Cu were calculated based on the present study and cited Cu data in the marine fishes from the literature; Table S4: Comparison of mean Zn concentrations (mg/kg dry weight (DW) and wet weight (WW)) in various species (15 species) of marine fishes reported in the literature; Table S5: Values of estimated daily intake (EDI), target hazard quotient (THQ), and estimated weekly intake (EWI) of Zn were calculated based on the present study and cited Zn data in the marine fishes from the literature.
