# Correction: Yap, C.K.; Al-Mutairi, K.A. Copper and Zinc Levels in Commercial Marine Fish from Setiu, East Coast of Peninsular Malaysia. *Toxics* 2022, *10*, 52

**DOI:** 10.3390/toxics10110649

**Published:** 2022-10-28

**Authors:** Chee Kong Yap, Khalid Awadh Al-Mutairi

**Affiliations:** 1 Department of Biology, Faculty of Science, Universiti Putra Malaysia (UPM), Serdang 43400, Selangor, Malaysia; 2 Department of Biology, Faculty of Science, University of Tabuk, Tabuk P.O. Box 741, Saudi Arabia

## Text Correction

The published article [1] with the following sentence for Section 1, paragraph 5 of the MS: “Aegean Seas of Turkey [46,47]”. However, it must be noted that the Aegean Sea of Greece for citation 47 must be included. This is because this work was conducted in the Greek Aegean Sea from a Greek research institute.

Therefore, it should be: “Aegean Sea/Eastern Mediterranean [46,47]”.

The authors state that the scientific conclusions are unaffected. This correction was approved by the Academic Editor. The original publication has also been updated.
