# Supplementary material for: Copper and Zinc Levels in Commercial Marine Fish from Setiu, East Coast of Peninsular Malaysia
Source: Toxics. 2022 Jan 24;10(2):52. doi: 10.3390/toxics10020052 (PMC8876149; doi:10.3390/toxics10020052)
Supplement: Supplementary file 1 [file toxics-10-00052-s001.zip › toxics-1539109-supplementary.pdf]

toxics

MDPI

Supplementary Materials: Copper and Zinc Levels in Commercial Marine Fish from Setiu, East Coast of Peninsular Malaysia

Chee Kong Yap and Khalid Awadh Al-Mutairi

Table S1. Description of the studied marine fishes, biometric features, water contents, and conversion factors.

Niche habitat	Site	Common name	Scientific name	N	Range of weight (g)	Range of length (cm)	Water content (%)	Conversion factor
			Family: Carangidae					
Reef-associated	1	Malabar trevally	*Carangoides malabaricus*	5	31–50	12.5–14.5	76	0.24
Reef-associated	1	Yellowtail scad	*Atule mate*	5	47–60	16.0–17.5	77	0.23
Reef-associated	1	Shortfin scad	*Decapterus macrosoma*	5	51–73	16.5–19.0	80	0.20
Reef-associated	1	Yellowstripe scad	*Selaroides leptolepis*	5	30–45	14.5–15.5	76	0.24
Reef-associated	1	Torpedo scad	*Megalaspis cordyla*	5	105–148	21.5–23.0	77	0.23
Reef-associated	1	Indian threadfish	*Alectis indica*	3	49–58	15.0–15.5	79	0.21
			Family: Sciaenidae					
Benthopelagic	2	Tigertooth croaker	*Otolithes ruber*	3	130–139	23.0–23.5	76	0.24
Demersal	2	Goatee croaker	*Dendrophysa russelli*	5	85–96	19.0–20.0	77	0.23
Demersal	2	Belanger's croaker	*Johnius belangerii*	3	62–74	16.0–17.5	77	0.23
			Family: Stromateidae					
Benthopelagic	1	Chinese silver pomfret	*Pampus chinensis*	3	138–149	18.0–18.5	81	0.19
			Family: Clupeidae					
Pelagic-neritic	1	Chacunda gizzard shard	*Anodontostoma chacunda*	5	46–53	14.5–16.0	77	0.23
			Family: Chirocentridae					
Reef-associated	1	Dorab wolf-herring	*Chirocentrus dorab*	5	120–148	32.0–34.5	77	0.23
Pelagic			Family: *Scrombidae*					
Pelagic-neritic	1	Indian mackerel	*Rastrelliger kanagurta*	5	58–86	17.0–18.5	74	0.26
Pelagic-neritic	1	Narrow-barred Spanish mackerel	*Scomberomorus commerson*	3	325–335	36.0–37.0	77	0.23
			Family: *Dasyatidae*					
Demersal	1	Dwarf whipray	*Himantura walga*	3	124–186	17.0–18.0	79	0.21
			Family: Nemipteridae					
Demersal	1	Ornate threadfin bream	*Nemipterus hexodon*	5	66–112	16.0–20.0	80	0.20
			Family: Lactariidae					
Pelagic-neritic	1	False trevally	*Lactarius lactarius*	5	47–61	15.5–17.0	79	0.21
			Family: Trichiuridae					
Benthopelagic	1	Largehead hairtail	*Trichiurus lepturus*	3	128–150	43.0–46.0	78	0.22
			Family: Ariidae					
Demersal	1	Spotted catfish	*Arius maculatus*	3	70–130	18.5–23.0	77	0.23

Table S2. Comparisons of mean Cu concentrations (mg/kg dry weight (DW) and wet weight (WW)) between the present study and reported studies (15 species) of marine fishes in literature.

No	Species	Location	DW	WW	References
1.	*Carangoides malabaricus*	Setiu	2.61	0.63	This study
		Andaman Sea	0.36	0.09	Kureishy *et al.* [1]
2	*Atule mate*	Setiu	7.83	1.80	This study
		Kuala Terengganu	3.32–6.67	1.53**	Ong *et al.* [2]
		Marine fish Peninsular Malaysia	-	0.92*	Wan Azmi *et al.* [3]
3	*Decapterus macrosoma*	Setiu	7.12	1.42	This study
		Langkawi	3.48	0.70	Agusa *et al.* [4]
		Kuala Terengganu	2.82–55.99	11.2**	Ong *et al.* [2]
		Marine fish Peninsular Malaysia	-	0.88*	Wan Azmi *et al.* [3]
		Gulf of Aqaba, Red Sea, Jordan	6.11	1.22	Khalaf *et al.* [5]
4	*Selaroides leptolepis*	Setiu	3.71	0.89	This study
		Marine fish Peninsular Malaysia	-	0.50*	Wan Azmi *et al.* [3]
		Port Dickson	2.46	0.59	Praveena and Lin [6]
		Pahang coastal waters	2.10	0.50	Kamaruzzaman and Wong [7]
		West coast of Peninsular Malaysia	-	1.41*	Nurnadia *et al.* [8]
5	*Megalaspis cordyla*	Setiu	4.47	1.03	This study
		Langkawi	2.21	0.51	Agusa *et al.* [4]
		Port Dickson	2.87	0.66	Agusa *et al.* [4]
		Cabang Tiga, Kelantan	2.53	0.58	Agusa *et al.* [4]
		Sihanouk Ville, Cambodia	2.48	0.57	Agusa *et al.* [4]
		Trat, Thailand	2.67	0.61	Agusa *et al.* [9]
		Prachuap Khiri Khan, Thailand	2.96	0.68	Agusa *et al.* [9]
		Khlong Yai, Thailand	4.06	0.93	Agusa *et al.* [9]
		Krabi, Thailand	3.57	0.82	Agusa *et al.* [9]
		Trang, Thailand	2.81	0.65	Agusa *et al.* [9]
		Smaut Sakrorn-1	4.39	1.01	Agusa *et al.* [9]
		Smaut Sakrorn-2	3.60	0.83	Agusa *et al.* [9]
		Kuala Kedah	3.36	0.77	Salam *et al.* [10]
		Tanjung Sepat, Selangor	25.69	5.91	Salam *et al.* [10]
		Marine fish Peninsular Malaysia	-	1.61*	Wan Azmi *et al.* [3]
		Port Dickson	1.40	0.32	Praveena and Lin [6]
		Pahang coastal waters	3.10	0.71	Kamaruzzaman and Ong [7]
		Mersing	1.51	0.35	Fathi *et al.* [11]
		Cochin coast, India	1.43	0.33	Nair *et al.* [12]
		West coast of Peninsular Malaysia	-	1.56*	Nurnadia *et al.* [8]
6	*Otolithes ruber*	Setiu	3.76	0.90	This study
		Khlong Yai, Thailand	1.94	0.47	Agusa *et al.* [9]
		Miri	12.4	2.98	Anandkumar *et al.* [13]
		Chabahar Bay, Makoran, Iran	2.40	0.58	Agah [14]
		Marine fish Peninsular Malaysia	-	0.29*	Wan Azmi *et al.* [3]
		North of Persian Gulf	8.46	2.03	Niri *et al.* [15]
		Kuala Tanjung coast, North Sumatra	-	0.19*	Simanjuntak *et al.* [16]
		Northwest coastal Karachi, Pakistan	-	0.36*	Raza *et al.* [17]
		Khuzestan shore, northwest of the Persian Gulf	4.09	0.98	Hosseini *et al.* [18]
		Kharg Island, Persian Gulf	-	0.98*	Abadi *et al.* [19]
7	*Dendrophysa russelli*	Setiu	1.50	0.35	This study
		Mumbai Harbor, India	1.65	0.38	Velusamy *et al.* [20]
8	*Johnius belangeri*	Setiu	2.25	0.52	This study
		Kapar	0.66	0.15	Bashir *et al.* [21]
		Mersing	0.95	0.22	Bashir *et al.* [21]
		Blanakan River Estuary, Indonesia	3.00	0.69	Takarina *et al.* [22]

		Kuala Tanjung coast, North Sumatra	-	0.11*	Simanjuntak *et al.* [16]
		Musa estuary, Iran	1.56	0.36	Abdollahpur Monikh *et al.* [23]
		Daya Bay's Fishery Resource Reserve, South China Sea	-	0.07*	Gu *et al.* [24]
9	*Pampus chinensis*	Setiu	1.50	0.29	This study
		Karachi Fish Harbour, Pakistan	1.05	0.20	Ahmed and Bat [25]
		Cox's Bazar, Bangladesh	0.20	0.04	Rakib *et al.* [26]
		Northwest coastal Karachi, Pakistan	-	0.30*	Raza *et al.* [17]
		Karnaphuli River estuary, Bangladesh	12.1	2.30	Ahmed *et al.* [27]
		South-Eastern Part of Bangladesh	16.2	3.08	Tasnim *et al.* [28]
10	*Anodontostama chacunda*	Setiu	3.73	0.86	This study
		Lada Bay, Panimbang, Indonesia	1.74	0.40	Agusa *et al.* [4]
		Bondet, Cirebon, Indonesia	1.47	0.34	Agusa *et al.* [9]
		Mengabang Telipot River, Kuala Terengganu	7.00	1.61	Kamaruzzaman *et al.* [29]
		Arabian Sea coasts of Pakistan	0.22–7.96	1.83**	Ahmed *et al.* [30]
11	*Chirocentrus dorab*	Setiu	2.25	0.52	This study
		Cochin coast, India	1.51	0.35	Nair *et al.* [12]
		Palk Bay, Southeastern India	-	1.14*	Arulkumar *et al.* [31]
		West coast of Peninsular Malaysia	-	0.99*	Nurnadia *et al.* [8]
12	*Rastrelliger kanagurta*	Setiu	5.56	1.45	This study
		Mersing	1.94	0.50	Agusa *et al.* [4]
		Lada Bay, Panimbang, Indonesia	1.09	0.28	Agusa *et al.* [4]
		Song Khla, Thailand	2.69	0.70	Agusa *et al.* [9]
		Ranong, Thailand	1.64	0.43	Agusa *et al.* [9]
		Saint Martin Island, Bangladesh	2.23	0.58	Baki *et al.* [32]
		Andaman Sea	-	0.96*	Kureishy *et al.* [1]
		Marine fish Peninsular Malaysia	-	0.97*	Wan Azmi *et al.* [3]
		Port Dickson	2.15	0.56	Praveena and Lin [6]
		Pahang coastal waters	2.00	0.52	Kamaruzzaman *et al.* [7]
		Cochin coast, India	2.10	0.55	Nair *et al.* [12]
		Palk Bay, Southeastern India	-	1.30*	Arulkumar *et al.* [31]
		Coastal waters off Kochi, India	2.75	0.72	Rejomon *et al.* [33]
		Langkawi Island	12.78	3.30	Irwandi and Farida [34]
		West coast of Peninsular Malaysia	-	0.89*	Nurnadia *et al.* [8]
		Kunduchi fish market in Dar es Salaam, Tanzania	9.17	2.38	Mziray and Kimirei [35]
13	*Scomberomorus commerson*	Setiu	1.86	0.43	This study
		Koh Kong, Cambodia	1.33	0.31	Agusa *et al.* [4]
		Marine fish Peninsular Malaysia	-	0.30*	Wan Azmi *et al.* [3]
		Langkawi Island	12.6	2.90	Irwandi and Farida [34]
		Coast of Karachi, Pakistan	5.52	1.27	Ahmed *et al.* [36]
		Zhongsha (Macclesfield) Fishing Ground, South China Sea	-	0.37*	Gu *et al.* [37]
14	*Trichiurus lepturus*	Setiu	2.99	0.66	This study
		Miri	9.00	1.98	Anandkumar *et al.* [13]
		Kutubdia Island, The Bay of Bengal	2.14-2.84	0.62**	Safiur Rahman *et al.* [38]
		Mumbai Harbor, India	2.11	0.46	Velusamy *et al.* [20]
15	*Arius maculatus*	Setiu	3.38	0.78	This study
		Mumbai Harbor, India	1.75	0.40	Velusamy *et al.* [20]
16	*Himantura walga*	Setiu	2.99	0.63	This study
17	*Nemipterus hexodon*	Setiu	2.61	0.52	This study
18	*Alectis indica*	Setiu	3.33	0.70	This study
19	*Lactarius lactarius*	Setiu	2.22	0.47	This study

Note: The data cited from the literature were recalculated for EDI and THQ based on fish consumption rate (100 g/person/day) and body weight of 62 kg for the adult Malaysian population, according to Nurul Izzah *et al.* [39]. The data which were originally reported on a dry weight basis were all converted into a wet weight basis based on the conversion factor for each species from this study. * data that were originally reported on a wet weight basis. ** The maximum value was selected for recalculation.

Table S3. Values of estimated daily intake (EDI), target hazard quotient (THQ), and estimated weekly intake (EWI) of Cu were calculated based on the present study and cited Cu data in the marine fishes from the literature.

No	Species	Location	EDI	THQ	EWI	References
1.	*Carangoides malabaricus*	Setiu	1.01	0.025	7.07	This study
		Andaman Sea	0.14	0.003	0.98	Kureishy *et al.* [1]
2	*Atule mate*	Setiu	2.90	0.073	20.33	This study
		Kuala Terengganu	2.47	0.062	17.32	Ong *et al.* [2]
		Marine fish Peninsular Malaysia	1.49	0.037	10.43	Wan Azmi *et al.* [3]
3	*Decapterus macrosoma*	Setiu	2.30	0.057	16.08	This study
		Langkawi	1.12	0.028	7.86	Agusa *et al.* [4]
		Kuala Terengganu	18.06	0.452	126.45	Ong *et al.* [2]
		Marine fish Peninsular Malaysia	1.41	0.035	9.90	Wan Azmi *et al.* [3]
		Gulf of Aqaba, Red Sea, Jordan	1.97	0.049	13.80	Khalaf *et al.* [5]
4	*Selaroides leptolepis*	Setiu	1.44	0.036	10.05	This study
		Marine fish Peninsular Malaysia	0.81	0.020	5.65	Wan Azmi *et al.* [3]
		Port Dickson	0.95	0.024	6.67	Praveena and Lin [6]
		Pahang coastal waters	0.81	0.020	5.69	Kamaruzzaman *et al.* [7]
		West coast of Peninsular Malaysia	2.27	0.057	15.92	Nurnadia *et al.* [8]
5	*Megalaspis cordyla*	Setiu	1.66	0.041	11.61	This study
		Langkawi	0.82	0.020	5.74	Agusa *et al.* [4]
		Port Dickson	1.06	0.027	7.45	Agusa *et al.* [4]
		Cabang Tiga, Kelantan	0.94	0.023	6.57	Agusa *et al.* [4]
		Sihanouk Ville, Cambodia	0.92	0.023	6.44	Agusa *et al.* [4]
		Trat, Thailand	0.99	0.025	6.93	Agusa *et al.* [9]
		Prachuap Khiri Khan, Thailand	1.10	0.027	7.69	Agusa *et al.* [9]
		Khlong Yai, Thailand	1.51	0.038	10.54	Agusa *et al.* [9]
		Krabi, Thailand	1.32	0.033	9.27	Agusa *et al.* [9]
		Trang, Thailand	1.04	0.026	7.30	Agusa *et al.* [9]
		Smaut Sakrorn-1	1.63	0.041	11.40	Agusa *et al.* [9]
		Smaut Sakrorn-2	1.34	0.033	9.35	Agusa *et al.* [9]
		Kuala Kedah	1.25	0.031	8.73	Salam *et al.* [10]
		Tanjung Sepat, Selangor	9.53	0.238	66.71	Salam *et al.* [10]
		Marine fish Peninsular Malaysia	2.60	0.065	18.22	Wan Azmi *et al.* [3]
		Port Dickson	0.52	0.013	3.64	Praveena and Lin [6]
		Pahang coastal waters	1.15	0.029	8.05	Kamaruzzaman *et al.* [7]
		Mersing	0.56	0.014	3.92	Fathi *et al.* [11]
		Cochin coast, India	0.53	0.013	3.71	Nair *et al.* [12]
		West coast of Peninsular Malaysia	2.52	0.063	17.61	Nurnadia *et al.* [8]
6	*Otolithes ruber*	Setiu	1.46	0.036	10.19	This study
		Khlong Yai, Thailand	0.75	0.019	5.26	Agusa *et al.* [9]
		Miri	4.80	0.120	33.60	Anandkumar *et al.* [13]
		Chabahar Bay, Makoran, Iran	0.93	0.023	6.50	Agah [14]
		Marine fish Peninsular Malaysia	0.47	0.012	3.27	Wan Azmi *et al.* [3]
		North of Persian Gulf	3.27	0.082	22.92	Niri *et al.* [15]
		Kuala Tanjung coast, North Sumatra	0.31	0.008	2.17	Simanjuntak *et al.* [16]
		Northwest coastal Karachi, Pakistan	0.58	0.015	4.06	Raza *et al.* [17]

		Khuzestan shore, northwest of the Persian Gulf.	1.58	0.040	11.08	Hosseini *et al.* [18]
		Kharg Island, Persian Gulf	1.58	0.040	11.06	Abadi *et al.* [19]
7	*Dendrophysa russelli*	Setiu	0.56	0.014	3.90	This study
		Mumbai Harbor, India	0.61	0.015	4.28	Velusamy *et al.* [20]
8	*Johnius belangeri*	Setiu	0.83	0.021	5.84	This study
		Kapar	0.24	0.006	1.71	Bashir *et al.* [21]
		Mersing	0.35	0.009	2.47	Bashir *et al.* [21]
		Blanakan River Estuary, Indonesia	1.11	0.028	7.79	Takarina *et al.* [22]
		Kuala Tanjung coast, North Sumatra	0.18	0.004	1.24	Simanjuntak *et al.* [16]
		Musa estuary, Iran	0.58	0.014	4.05	Abdolahpur Monikh *et al.* [23]
		Daya Bay's Fishery Resource Reserve, South China Sea	0.11	0.003	0.79	Gu *et al.* [24]
9	*Pampus chinensis*	Setiu	0.46	0.011	3.22	This study
		Karachi Fish Harbour, Pakistan	0.32	0.008	2.25	Ahmed and Bat [36]
		Cox's Bazar, Bangladesh	0.06	0.002	0.43	Rakib *et al.* [26]
		Northwest coastal Karachi, Pakistan	0.48	0.012	3.39	Raza *et al.* [17]
		Karnaphuli River estuary, Bangladesh	3.71	0.093	25.96	Ahmed *et al.* [27]
		South-Eastern Part of Bangladesh	4.96	0.124	34.75	Tasnim *et al.* [28]
10	*Anodontostama chacunda*	Setiu	1.38	0.035	9.69	This study
		Lada Bay, Panimbang, Indonesia	0.65	0.016	4.52	Agusa *et al.* [4]
		Bondet, Cirebon, Indonesia	0.55	0.014	3.82	Agusa *et al.* [9]
		Mengabang Telipot River, Kuala Terengganu	2.60	0.065	18.18	Kamaruzzaman *et al.* [29]
		Arabian Sea coasts of Pakistan	2.95	0.074	20.67	Ahmed *et al.* [30]
11	*Chirocentrus dorab*	Setiu	0.83	0.021	5.84	This study
		Cochin coast, India	0.56	0.014	3.92	Nair *et al.* [12]
		Palk Bay, Southeastern India	1.84	0.046	12.87	Arulkumar *et al.* [31]
		West coast of Peninsular Malaysia	1.60	0.040	11.18	Nurnadia *et al.* [8]
12	*Rastrelliger kanagurta*	Setiu	2.33	0.058	16.32	This study
		Mersing (1998) (N = 5)	0.81	0.020	5.69	Agusa *et al.* [4]
		Lada Bay, Panimbang, Indonesia	0.46	0.011	3.20	Agusa *et al.* [4]
		Song Khla, Thailand	1.13	0.028	7.90	Agusa *et al.* [9]
		Ranong, Thailand	0.69	0.017	4.81	Agusa *et al.* [9]
		Saint Martin Island, Bangladesh	0.94	0.023	6.55	Baki *et al.* [32]
		Andaman Sea	1.55	0.039	10.84	Kureishy *et al.* [1]
		Marine fish Peninsular Malaysia	1.57	0.039	10.96	Wan Azmi *et al.* [3]
		Port Dickson	0.90	0.023	6.31	Praveena and Lin [6]
		Pahang coastal waters	0.84	0.021	5.87	Kamaruzzaman *et al.* [7]
		Cochin coast, India	0.88	0.022	6.16	Nair *et al.* [12]
		Palk Bay, Southeastern India	2.10	0.052	14.68	Arulkumar *et al.* [31]
		Coastal waters off Kochi, India	1.15	0.029	8.07	Rejomon *et al.* [33]
		Langkawi Island	5.32	0.133	37.22	Irwandi and Farida [34]
		West coast of Peninsular Malaysia	1.44	0.036	10.05	Nurnadia *et al.* [8]
		Kunduchi fish market in Dar es Salaam, Tanzania	3.85	0.096	26.92	Mziray and Kimirei [35]
13	*Scomberomorus commerson*	Setiu	0.69	0.017	4.83	This study
		Koh Kong, Cambodia	0.49	0.012	3.45	Agusa *et al.* [4]
		Marine fish Peninsular Malaysia	0.49	0.012	3.40	Wan Azmi *et al.* [3]

		Langkawi Island	4.67	0.117	32.72	Irwandi and Farida [34]
		Coast of Karachi, Pakistan	2.05	0.051	14.33	Ahmed *et al.* [36]
		Zhongsha (Macclesfield) Fishing Ground, South China Sea	0.60	0.015	4.18	Gu *et al.* [37]
14	*Trichiurus lepturus*	Setiu	1.06	0.027	7.43	This study
		Miri	3.19	0.080	22.35	Anandkumar *et al.* [13]
		Kutubdia Island, The Bay of Bengal	1.01	0.025	7.05	Rahman *et al.* [38]
		Mumbai Harbor, India	0.75	0.019	5.24	Velusamy *et al.* [20]
15	*Arius maculatus*	Setiu	1.25	0.031	8.78	This study
		Mumbai Harbor, India	0.65	0.016	4.54	Velusamy *et al.* [20]
16	*Himantura walga*	Setiu	1.01	0.025	7.09	This study
17	*Nemipterus hexodon*	Setiu	0.84	0.021	5.89	This study
18	*Alectis indica*	Setiu	1.13	0.028	7.90	This study
19	*Lactarius lactarius*	Setiu	0.75	0.019	5.26	This study

Note: PTWIs for a 62 kg adult is equivalent to 217 mg/week for Cu.

Table S4. Comparison of mean Zn concentrations (mg/kg dry weight (DW) and wet weight (WW)) in various species (15 species) of marine fishes reported in the literature.

No	Species	Location	DW	WW	References
1.	*Carangoides malabaricus*	Setiu	35.96	8.63	This study
		Andaman Sea	-	4.74*	Kureishy *et al.* [1]
2	*Atule mate*	Setiu	61.17	14.07	This study
		Kuala Terengganu	14–29.2	6.72**	Ong *et al.* [2]
		Marine fish Peninsular Malaysia	-	8.49*	Wan Azmi et al. [3]
3	*Decapterus macrosoma*	Setiu	54.85	10.97	This study
		Langkawi	29.1	5.82	Agusa *et al.* [4]
		Kuala Terengganu	10.4-42.3	8.46**	Ong *et al.* [2]
		Marine fish Peninsular Malaysia	-	15.90*	Wan Azmi et al. [3]
		Gulf of Aqaba, Red Sea, Jordan	20.3	4.06	Khalaf *et al.* [5]
4	*Selaroides leptolepis*	Setiu	58.78	14.11	This study
		Marine fish Peninsular Malaysia	-	7.20*	Wan Azmi et al. [3]
		Pahang coastal waters	11.0	2.64	Kamaruzzaman *et al.* [7]
		West coast of Peninsular Malaysia	-	4.49*	Nurnadia *et al.* [8]
5	*Megalaspis cordyla*	Setiu	45.74	10.52	This study
		Langkawi	17.9	4.12	Agusa *et al.* [4]
		Port Dickson	27.2	6.26	Agusa *et al.* [4]
		Cabang Tiga, Kelantan	28.3	6.51	Agusa *et al.* [4]
		Sihanouk Ville, Cambodia	27.9	6.42	Agusa *et al.* [9]
		Trat, Thailand	35.2	8.10	Agusa *et al.* [9]
		Prachuap Khiri Khan, Thailand	29.3	6.74	Agusa *et al.* [9]
		Khlong Yai, Thailand	23.6	5.43	Agusa *et al.* [9]
		Krabi, Thailand	20.4	4.69	Agusa *et al.* [9]
		Trang, Thailand	22.7	5.22	Agusa *et al.* [9]
		Smaut Sakrorn-1	21.3	4.90	Agusa *et al.* [9]
		Smaut Sakrorn-2	22.0	5.06	Agusa *et al.* [9]
		Marine fish Peninsular Malaysia	-	7.00*	Wan Azmi et al. [3]
		Pahang coastal waters	10.0	2.30	Kamaruzzaman *et al.* [7]
		Mersing	21.1	4.85	Fathi *et al.* [11]
		Karachi Fish Harbor of Pakistan	-	3.00	Ahmed and Bat [40]
		Cochin coast, India	19.3	4.44	Nair *et al.* [12]

		West coast of Peninsular Malaysia	-	3.99*	Nurnadia *et al.* [8]
6	*Otolithes ruber*	Setiu	30.2	7.26	This study
		Khlong Yai, Thailand	23.9	5.74	Agusa *et al.* [9]
		Miri	16.9	4.06	Anandkumar *et al.* [13]
		Chabahar Bay, Makoran, Iran	4.70	1.13	Agah [14]
		Marine fish Peninsular Malaysia	-	4.34*	Wan Azmi et al. [3]
		The northern part of the Hormuz strait (Persian Gulf)	23.3	5.58	Janadeleh and Jahangiri [41]
		Kuala Tanjung coast, North Sumatra	-	7.69*	Simanjuntak *et al.* [16]
		Southwest coast of Peninsular Malaysia	5.87	1.41	Kamaruzzaman *et al.* [42]
		Kharg Island, Persian Gulf	-	7.04*	Abadi *et al.* [19]
7	*Dendrophysa russelli*	Setiu	41.96	9.65	This study
		Mumbai Harbor, India	38.69	8.90	Velusamy *et al.* [20]
8	*Johnius belangeri*	Setiu	26.77	6.16	This study
		Kapar	18.27	4.20	Bashir *et al.* [21]
		Mersing	13.12	3.02	Bashir *et al.* [21]
		Blanakan River Estuary, Indonesia	29.0	6.67	Takarina *et al.* [22]
		Daya Bay’s Fishery Resource Reserve, South China Sea	-	4.57*	Gu *et al.* [24]
		Kuala Tanjung coast, North Sumatra	-	7.23*	Simanjuntak *et al.* [16]
9	*Pampus chinensis*	Setiu	32.59	6.19	This study
		Karachi Fish Harbour, Pakistan	-	1.67*	Ahmed and Bat [36]
		Kalimati fish market (Kathmandu)	25.4	4.83	Paudel *et al.* [43]
		Cox's Bazar, Bangladesh	68.7	13.05	Rakib *et al.* [26]
		South-Eastern Part of Bangladesh	8.00	1.52	Tasnim *et al.* [28]
10	*Anodontostama chacunda*	Setiu	42.67	9.81	This study
		Lada Bay, Panimbang, Indonesia	28.0	6.44	Agusa *et al.* [9]
		Bondet, Cirebon, Indonesia	27.1	6.23	Agusa *et al.* [9]
		Mengabang Telipot River, Kuala Terengganu	5.00	1.15	Kamaruzzaman *et al.* [29]
		Arabian Sea coasts of Pakistan	8.24–56.23	12.9**	Ahmed *et al.* [30]
11	*Chirocentrus dorab*	Setiu	31.0	7.14	This study
		Cochin coast, India	6.56	1.51	Nair *et al.* [12]
		Palk Bay, Southeastern India	29.8	6.85	Arulkumar *et al.* [31]
		Southwest coast of Peninsular Malaysia	3.32	0.76	Kamaruzaman *et al.* [42]
		West coast of Peninsular Malaysia	-	3.40*	Nurnadia *et al.* [8]
12	*Rastrelliger kanagurta*	Setiu	80.49	20.93	This study
		Mersing	23.2	6.03	Agusa *et al.* [4]
		Lada Bay, Panimbang, Indonesia	15.0	3.90	Agusa *et al.* [9]
		Song Khla, Thailand	50.7	13.18	Agusa *et al.* [9]
		Ranong, Thailand	21.1	5.49	Agusa *et al.* [9]
		Saint Martin Island, Bangladesh	9.21	2.39	Baki *et al.* [32]
		Andaman Sea	-	6.09*	Kureishy *et al.* [1]
		Marine fish Peninsular Malaysia	-	9.08*	Wan Azmi et al. [3]
		Pahang coastal waters	24.0	6.24	Kamaruzzaman *et al.* [7]

		Cochin coast, India	15.0	3.90	Nair *et al.* [12]
		Palk Bay, Southeastern India	29.3	7.62	Arulkumar *et al.* [31]
		Langkawi Island	38.9	10.13	Irwandi and Farida [34]
		West coast of Peninsular Malaysia	-	4.64	Nurnadia *et al.* [8]
		Coastal waters off Kochi, India	37.4	9.72	Rejomon *et al.* [33]
		Kunduchi fish market in Dar es Salaam, Tanzania	104	27.0	Mziray and Kimirei [35]
13	*Scomberomorus commerson*	Setiu	49.6	11.41	This study
		Koh Kong, Cambodia	17.5	4.03	Agusa *et al.* [9]
		Marine fish Peninsular Malaysia	-	4.69*	Wan Azmi et al. [3]
		Langkawi Island	37.2	8.56	Irwandi and Farida [34]
		Coast of Karachi, Pakistan	9.43	2.17	Ahmed *et al.* [36]
		Zhongsha (Macclesfield) Fishing Ground, South China Sea	-	4.24*	Gu *et al.* [37]
14	*Trichiurus lepturus*	Setiu	24.14	5.31	This study
		Miri	25.3	5.57	Anandkumar *et al.* [13]
		Kutubdia Island, The Bay of Bengal	23.04–27.34	6.01**	Rahman *et al.* [38]
		Mumbai Harbor, India	42.34	9.31	Velusamy *et al.* [20]
15	*Arius maculatus*	Setiu	33.99	7.82	This study
		Mumbai Harbor, India	55.3	12.72	Velusamy *et al.* [20]
16	*Himantura walga*	Setiu	28.11	5.90	This study
17	*Nemipterus hexodon*	Setiu	26.45	5.29	This study
18	*Alectis indica*	Setiu	27.89	5.86	This study
19	*Lactarius lactarius*	Setiu	32.62	6.85	This study

Note: The data cited from the literature were recalculated for EDI and THQ based on fish consumption rate (100 g/person/day) and body weight of 62 kg for the adult Malaysian population, according to Nurul Izzah *et al.* [39]. The data which were originally reported on a dry weight basis were all converted into a wet weight basis based on the conversion factor for each species from this study. * data that were originally reported on a wet weight basis. ** The maximum value was selected for recalculation.

Table S5. Values of estimated daily intake (EDI), target hazard quotient (THQ), and estimated weekly intake (EWI) of Zn were calculated based on the present study and cited Zn data in the marine fishes from the literature.

No	Species	Location	EDI	THQ	EWI	References
1.	*Carangoides malabaricus*	Setiu	13.92	0.046	97.4	This study
		Andaman Sea	7.65	0.025	53.5	Kureishy *et al.* [1]
2	*Atule mate*	Setiu	22.69	0.076	158.8	This study
		Kuala Terengganu	10.83	0.036	75.8	Ong *et al.* [2]
		Marine fish Peninsular Malaysia	13.69	0.046	95.9	Wan Azmi et al. [3]
3	*Decapterus macrosoma*	Setiu	17.69	0.059	123.9	This study
		Langkawi	9.39	0.031	65.7	Agusa *et al.* [4]
		Kuala Terengganu	13.65	0.045	95.5	Ong *et al.* [2]
		Marine fish Peninsular Malaysia	25.65	0.085	179.5	Wan Azmi et al. [3]
		Gulf of Aqaba, Red Sea, Jordan	6.55	0.022	45.8	Khalaf *et al.* [5]
4	*Selaroides leptolepis*	Setiu	22.75	0.076	159.3	This study
		Marine fish Peninsular Malaysia	11.61	0.039	81.3	Wan Azmi et al. [3]
		Pahang coastal waters	4.26	0.014	29.8	Kamaruzzaman *et al.* [7]
		West coast of Peninsular Malaysia	7.24	0.024	50.7	Nurnadia *et al.* [8]
5	*Megalaspis cordyla*	Setiu	16.97	0.057	118.8	This study

		Langkawi	6.64	0.022	46.5	Agusa *et al.* [4]
		Port Dickson	10.09	0.034	70.6	Agusa *et al.* [4]
		Cabang Tiga, Kelantan	10.50	0.035	73.5	Agusa *et al.* [4]
		Sihanouk Ville, Cambodia	10.35	0.035	72.5	Agusa *et al.* [9]
		Trat, Thailand	13.06	0.044	91.4	Agusa *et al.* [9]
		Prachuap Khiri Khan, Thailand	10.87	0.036	76.1	Agusa *et al.* [9]
		Khlong Yai, Thailand	8.75	0.029	61.3	Agusa *et al.* [9]
		Krabi, Thailand	7.57	0.025	53.0	Agusa *et al.* [9]
		Trang, Thailand	8.42	0.028	58.9	Agusa *et al.* [9]
		Smaut Sakrorn-1	7.90	0.026	55.3	Agusa *et al.* [9]
		Smaut Sakrorn-2	8.16	0.027	57.1	Agusa *et al.* [9]
		Marine fish Peninsular Malaysia	11.29	0.038	79.0	Wan Azmi et al. [3]
		Pahang coastal waters	3.71	0.012	26.0	Kamaruzzaman *et al.* [7]
		Mersing	7.83	0.026	54.8	Fathi *et al.* [11]
		Karachi Fish Harbor of Pakistan	4.84	0.016	33.9	Ahmed and Bat [40]
		Cochin coast, India	7.16	0.024	50.1	Nair *et al.* [12]
		West coast of Peninsular Malaysia	6.44	0.021	45.0	Nurnadia *et al.* [8]
6	*Otolithes ruber*	Setiu	11.71	0.039	81.9	This study
		Khlong Yai, Thailand	9.25	0.031	64.8	Agusa *et al.* [9]
		Miri	6.54	0.022	45.8	Anandkumar *et al.* [13]
		Chabahar Bay, Makoran, Iran	1.82	0.006	12.7	Agah [14]
		Marine fish Peninsular Malaysia	7.00	0.023	49.0	Wan Azmi et al. [3]
		The northern part of the Hormuz strait (Persian Gulf)	9.00	0.030	63.0	Janadeleh and Jahangiri [41]
		Kuala Tanjung coast, North Sumatra	12.40	0.041	86.8	Simanjuntak *et al.* [16]
		Southwest coast of Peninsular Malaysia	2.27	0.008	15.9	Kamaruzzaman *et al.* [42]
		Kharg Island, Persian Gulf	11.35	0.038	79.5	Abadi *et al.* [19]
7	*Dendrophysa russelli*	Setiu	15.57	0.052	109.0	This study
		Mumbai Harbor, India	14.35	0.048	100.5	Velusamy *et al.* [20]
8	*Johnius belangeri*	Setiu	9.93	0.033	69.5	This study
		Kapar	6.78	0.023	47.4	Bashir *et al.* [21]
		Mersing	4.87	0.016	34.1	Bashir *et al.* [21]
		Blanakan River Estuary, Indonesia	10.76	0.036	75.3	Takarina *et al.* [22]
		Daya Bay's Fishery Resource Reserve, South China Sea	7.37	0.025	51.6	Gu *et al.* [24]
		Kuala Tanjung coast, North Sumatra	11.66	0.039	81.6	Simanjuntak *et al.* [16]
9	*Pampus chinensis*	Setiu	9.99	0.033	69.9	This study
		Karachi Fish Harbour, Pakistan	2.69	0.009	18.9	Ahmed and Bat [36]
		Kalimati fish market (Kathmandu)	7.78	0.026	54.5	Paudel *et al.* [43]
		Cox's Bazar, Bangladesh	21.05	0.070	147.4	Rakib *et al.* [26]
		South-Eastern Part of Bangladesh	2.45	0.008	17.2	Tasnim *et al.* [28]
10	*Anodontostama chacunda*	Setiu	15.83	0.053	110.8	This study

		Lada Bay, Panimbang, Indonesia	10.39	0.035	72.7	Agusa *et al.* [9]
		Bondet, Cirebon, Indonesia	10.05	0.034	70.4	Agusa *et al.* [9]
		Mengabang Telipot River, Kuala Terengganu	1.85	0.006	13.0	Kamaruzzaman *et al.* [29]
		Arabian Sea coasts of Pakistan	20.85	0.069	145.9	Ahmed *et al.* [30]
11	*Chirocentrus dorab*	Setiu	11.51	0.038	80.6	This study
		Cochin coast, India	2.43	0.008	17.0	Nair *et al.* [12]
		Palk Bay, Southeastern India	11.05	0.037	77.4	Arulkumar *et al.* [31]
		Southwest coast of Peninsular Malaysia	1.23	0.004	8.6	Kamaruzaman *et al.* [42]
		West coast of Peninsular Malaysia	5.48	0.018	38.4	Nurnadia *et al.* [8]
12	*Rastrelliger kanagurta*	Setiu	33.75	0.113	236.3	This study
		Mersing	9.73	0.032	68.1	Agusa *et al.* [4]
		Lada Bay, Panimbang, Indonesia	6.29	0.021	44.0	Agusa *et al.* [9]
		Song Khla, Thailand	21.26	0.071	148.8	Agusa *et al.* [9]
		Ranong, Thailand	8.85	0.029	61.9	Agusa *et al.* [9]
		Saint Martin Island, Bangladesh	3.86	0.013	27.0	Baki *et al.* [32]
		Andaman Sea	9.82	0.033	68.8	Kureishy *et al.* [1]
		Marine fish Peninsular Malaysia	14.65	0.049	102.5	Wan Azmi et al. [3]
		Pahang coastal waters	10.06	0.034	70.5	Kamaruzzaman *et al.* [7]
		Cochin coast, India	6.29	0.021	44.0	Nair *et al.* [12]
		Palk Bay, Southeastern India	12.29	0.041	86.0	Arulkumar *et al.* [31]
		Langkawi Island	16.33	0.054	114.3	Irwandi and Farida [34]
		West coast of Peninsular Malaysia	7.48	0.025	52.4	Nurnadia *et al.* [8]
		Coastal waters off Kochi, India	15.68	0.052	109.8	Rejomon *et al.* [33]
		Kunduchi fish market in Dar es Salaam, Tanzania	43.61	0.145	305.3	Mziray and Kimirei [35]
13	*Scomberomorus commerson*	Setiu	18.41	0.061	128.9	This study
		Koh Kong, Cambodia	6.49	0.022	45.4	Agusa *et al.* [9]
		Marine fish Peninsular Malaysia	7.56	0.025	53.0	Wan Azmi et al. [3]
		Langkawi Island	13.81	0.046	96.7	Irwandi and Farida [34]
		Coast of Karachi, Pakistan	3.50	0.012	24.5	Ahmed *et al.* [36]
		Zhongsha (Macclesfield) Fishing Ground, South China Sea	6.84	0.023	47.9	Gu *et al.* [37]
14	*Trichiurus lepturus*	Setiu	8.57	0.029	60.0	This study
		Miri	8.98	0.030	62.8	Anandkumar *et al.* [13]
		Kutubdia Island, The Bay of Bengal	9.69	0.032	67.8	Rahman *et al.* [38]
		Mumbai Harbor, India	15.02	0.050	105.2	Velusamy *et al.* [20]
15	*Arius maculatus*	Setiu	12.61	0.042	88.3	This study
		Mumbai Harbor, India	20.51	0.068	143.6	Velusamy *et al.* [20]
16	*Himantura walga*	Setiu	9.52	0.032	66.6	This study
17	*Nemipterus hexodon*	Setiu	8.53	0.028	59.7	This study
18	*Alectis indica*	Setiu	9.45	0.031	66.1	This study
19	*Lactarius lactarius*	Setiu	11.05	0.037	77.3	This study

Note: PTWIs for a 62 kg adult is equivalent to 434 mg/week for Zn.

References

1. Kureishy, T.W.; Sanzgiry, S.; Braganca, A. Some Heavy Metals in Fishes from the Andaman Sea. *Indian J. Mar. Sci.* **1981**, *10*, 303–307.
2. Ong, M.C.; Abd Aziz, N.; M Shazili, N.A.; Yunus, K. Selected Heavy Metals Content in Commercial Fishes at Different Season Landed at Fisheries Development Authority of Malaysia Complex (LKIM) Complex, Kuala Terengganu, Malaysia. *J. Sustain. Sci. Manag.* **2018**, *13*, 29–38.
3. Wan Azmi, W.N.F.; Nurul Izzah, A.; Wan Mahiyuddin, W.R. Heavy Metal Levels and Risk Assessment from Consumption of Marine Fish in Peninsular Malaysia. *J. Environ. Prot.* **2019**, *10*, 1450–1471, doi:10.4236/jep.2019.1011086.
4. Agusa, T.; Kunito, T.; Yasunaga, G.; Iwata, H.; Subramanian, A.; Ismail, A.; Tanabe, S. Concentrations of Trace Elements in Marine Fish and Its Risk Assessment in Malaysia. *Mar. Pollut. Bull.* **2005**, *51*, 896–911, doi:10.1016/j.marpolbul.2005.06.007.
5. Khalaf, M.; Al-Najjar, T.; Alawi, Dr.M.; A.Disi, A. Levels of Trace Metals in Three Fish Species Decapterus Macrellus, Decapterus Macrosomos and Decapterus Russelli of the Family Carangidae from the Gulf of Aqaba, Read Sea, Jordan. *Nat. Sci.* **2012**, *4*, 362–367, doi:10.4236/ns.2012.46050.
6. Praveena, S.; Lin, C.L.S. Assessment of Heavy Metal in Self-Caught Saltwater Fish from Port Dickson Coastal Water, Malaysia. *Sains Malays.* **2015**, *44*, 91–99, doi:10.17576/jsm-2015-4401-13.
7. Kamaruzzaman, Y.; Ong, M.C.; Rina, S.Z. Concentration of Zn, Cu and Pb in Some Selected Marine Fishes of the Pahang Coastal Waters, Malaysia. *Am. J. Appl. Sci.* **2010**, *7*, 309–314, doi:10.3844/ajassp.2010.309.314.
8. Nurnadia, A.A.; Azrina, A.; Amin, I.; Mohd Yunus, A.S.; Effendi Halmi, M. Mineral Contents of Selected Marine Fish and Shellfish from the West Coast of Peninsular Malaysia. *Int. Food Res. J.* **2013**, *20*, 431–437.
9. Agusa, T.; Kunito, T.; Sudaryanto, A.; Monirith, I.; Kan-Atireklap, S.; Iwata, H.; Ismail, A.; Sanguansin, J.; Muchtar, M.; Tana, T.S.; et al. Exposure Assessment for Trace Elements from Consumption of Marine Fish in Southeast Asia. *Environ. Pollut. Barking Essex 1987* **2007**, *145*, 766–777, doi:10.1016/j.envpol.2006.04.034.
10. Salam, M.A.; Dayal, S.R.; Siddiqua, S.A.; Muhib, M.I.; Bhowmik, S.; Kabir, M.M.; Rak, A.A.E.; Srzednicki, G. Risk Assessment of Heavy Metals in Marine Fish and Seafood from Kedah and Selangor Coastal Regions of Malaysia: A High-Risk Health Concern for Consumers. *Environ. Sci. Pollut. Res. Int.* **2021**, *28*, 55166–55175, doi:10.1007/s11356-021-14701-z.
11. Fathi, B.; Othman, M.; Mazlan, A.G.; Idris, G.; Arshad, A.; Amin, S.M.N.; Simon, D. Trace Metals in Muscle, Liver and Gill Tissues of Marine Fishes from Mersing, Eastern Coast of Peninsular Malaysia: Concentration and Assessment of Human Health Risk. *Asian J. Anim. Vet. Adv.* **2013**, *8*, 227–236, doi:10.3923/ajava.2013.227.236.
12. Nair, M.; Balachandran, K.K.; Sankaranarayanan, V.N.; Joseph, T. Heavy Metals in Fishes from Coastal Waters of Cochin, Southwest Coast of India. *Int. J. Mar. Sci.* **1997**, *26*, 98–100.
13. Anandkumar, R.N.; Prabakaran, K.; Chua, H.B.; Rajaram, D.R. Human Health Risk Assessment and Bioaccumulation of Trace Metals in Fish Species Collected from the Miri Coast, Sarawak, Borneo. *Mar. Pollut. Bull.* **2018**, *133*, 655–663, doi:10.1016/j.marpolbul.2018.06.033.
14. Agah, H. Ecological Risk Assessment of Heavy Metals in Sediment, Fish, and Human Hair from Chabahar Bay, Makoran, Iran. *Mar. Pollut. Bull.* **2021**, *169*, 112345, doi:10.1016/j.marpolbul.2021.112345.
15. Niri, A.S.; Sharifian, S.; Ahmadi, R. Assessment of Metal Accumulation in Two Fish Species (Tenualosa Ilisha and Otolithes Ruber), Captured from the North of Persian Gulf. *Bull. Environ. Contam. Toxicol.* **2015**, *94*, 71–76, doi:10.1007/s00128-014-1429-9.
16. Simanjuntak, C.; Djumanto, D.; Rahardjo, M.; Zahid, A. Assessment of Heavy Metals (Al, Zn, Cu, Cd, Pb and Hg) in Demersal Fishes of Kuala Tanjung Coast, North Sumatera. In Proceedings of the FAPERIKA-UNRI; Riau University: Indonesia, December 1 2012; pp. 178–196.

17. Raza, R.; Sayeed, S.A.; Siddiqi, R.; Naz, S. Trace Metal Contents in Selected Marine Fish Species of Northwest Coastal Area of Karachi, Pakistan. *J. Chem. Soc. Pak.* **2003**, *25*, 313–316.

18. Hosseini, M.; Nabavi, S.M.B.; Nabavi, S.N.; Pour, N.A. Heavy Metals (Cd, Co, Cu, Ni, Pb, Fe, and Hg) Content in Four Fish Commonly Consumed in Iran: Risk Assessment for the Consumers. *Environ. Monit. Assess.* **2015**, *187*, 237, doi:10.1007/s10661-015-4464-z.

19. Abadi, D.R.V.; Dobaradaran, S.; Nabipour, I.; Lamani, X.; Ravanipour, M.; Tahmasebi, R.; Nazmara, S. Comparative Investigation of Heavy Metal, Trace, and Macro Element Contents in Commercially Valuable Fish Species Harvested off from the Persian Gulf. *Environ. Sci. Pollut. Res. Int.* **2015**, *22*, 6670–6678, doi:10.1007/s11356-014-3852-1.

20. Velusamy, A.; Satheesh Kumar, P.; Ram, A.; Chinnadurai, S. Bioaccumulation of Heavy Metals in Commercially Important Marine Fishes from Mumbai Harbor, India. *Mar. Pollut. Bull.* **2014**, *81*, 218–224, doi:10.1016/j.marpolbul.2014.01.049.

21. Bashir, F.; Othman, M.; A.G., M.; M Rahim, S.; Das, S. Heavy Metal Concentration in Fishes from the Coastal Waters of Kapar and Mersing, Malaysia. *Turk. J. Fish. Aquat. Sci.* **2013**.

22. Takarina, N.D.; Purwiyanto, A.I.S.; Suteja, Y. Cadmium (Cd), Copper (Cu), and Zinc (Zn) Levels in Commercial and Non-Commercial Fishes in the Blanakan River Estuary, Indonesia: A Preliminary Study. *Mar. Pollut. Bull.* **2021**, *170*, 112607, doi:10.1016/j.marpolbul.2021.112607.

23. Abdolahpur Monikh, F.; Safahieh, A.; Savari, A.; Ronagh, M.T.; Doraghi, A. The Relationship between Heavy Metal (Cd, Co, Cu, Ni and Pb) Levels and the Size of Benthic, Benthopelagic and Pelagic Fish Species, Persian Gulf. *Bull. Environ. Contam. Toxicol.* **2013**, *90*, 691–696, doi:10.1007/s00128-013-0986-7.

24. Gu, Y.-G.; Huang, H.-H.; Lin, Q. Concentrations and Human Health Implications of Heavy Metals in Wild Aquatic Organisms Captured from the Core Area of Daya Bay's Fishery Resource Reserve, South China Sea. *Environ. Toxicol. Pharmacol.* **2016**, *45*, 90–94, doi:10.1016/j.etap.2016.05.022.

25. Ahmed, Q.; Bat, L. Potential Risk of Some Heavy Metals in Pampus Chinensis (Euphrasen) Chinese Silver Pomfret Stromateidae Collected from Karachi Fish Harbour, Pakistan. *Int. J. Mar. Sci.* **2015**, *555*, 1–5, doi:10.5376/ijms.2015.05.0021.

26. Rakib, M.R.J.; Jolly, Y.N.; Enyoh, C.E.; Khandaker, M.U.; Hossain, M.B.; Akther, S.; Alsubaie, A.; Almalki, A.S.A.; Bradley, D.A. Levels and Health Risk Assessment of Heavy Metals in Dried Fish Consumed in Bangladesh. *Sci. Rep.* **2021**, *11*, 14642, doi:10.1038/s41598-021-93989-w.

27. Ahmed, A.S.S.; Sultana, S.; Habib, A.; Ullah, H.; Musa, N.; Hossain, M.B.; Rahman, M.M.; Sarker, M.S.I. Bioaccumulation of Heavy Metals in Some Commercially Important Fishes from a Tropical River Estuary Suggests Higher Potential Health Risk in Children than Adults. *PloS One* **2019**, *14*, e0219336, doi:10.1371/journal.pone.0219336.

28. Tasnim, K.T.; Akter, S.; Shoeb, M.; Farha, W. Fatty Acids Composition and Heavy Metals in Marine Fish Samples from the South-Eastern Part of Bangladesh. *Org. Chem. Plus* **2020**, *1*, 46–51, doi:10.37256/ocp.122020457.

29. Kamaruzzaman, B.Y.; Ong, M.C.; Jalal, K.C.A. Levels of Copper, Zinc and Lead in Fishes of Mengabang Telipot River,Terengganu, Malaysia. *J. Biol. Sci.* **2008**, *8*, doi:10.3923/jbs.2008.1181.1186.

30. Ahmed, Q.; Bat, L.; Mohammad, Q. Bioaccumulation of Nine Heavy Metals in Some Tissues of Anodontostoma Chacunda (Hamilton, 1822) in the Arabian Sea Coasts of Pakistan. *Nat. Eng. Sci.* **2017**, *2*, 79–92, doi:10.28978/nesciences.349296.

31. Arulkumar, A.; Paramasivam, S.; Rajaram, R. Toxic Heavy Metals in Commercially Important Food Fishes Collected from Palk Bay, Southeastern India. *Mar. Pollut. Bull.* **2017**, *119*, 454–459, doi:10.1016/j.marpolbul.2017.03.045.

32. Baki, M.A.; Hossain, M.M.; Akter, J.; Quraishi, S.B.; Haque Shojib, M.F.; Atique Ullah, A.K.M.; Khan, M.F. Concentration of Heavy Metals in Seafood (Fishes, Shrimp, Lobster and Crabs) and Human Health Assessment in Saint Martin Island, Bangladesh. *Ecotoxicol. Environ. Saf.* **2018**, *159*, 153–163, doi:10.1016/j.ecoenv.2018.04.035.

33. Rejomon, G.; Nair, M.; Joseph, T. Trace Metal Dynamics in Fishes from the Southwest Coast of India. *Environ. Monit. Assess.* **2010**, *167*, 243–255, doi:10.1007/s10661-009-1046-y.

34. Irwandi, J.; Farida, M. Mineral and Heavy Metal Contents of Marine Fin Fish in Langkawi Island, Malaysia. *Int. Food Res. J.* **2009**, *16*.
35. Mziray, P.; Kimirei, I. Bioaccumulation of Heavy Metals in Marine Fishes (Siganus Sutor, Lethrinus Harak, and Rastrelliger Kanagurta) from Dar Es Salaam Tanzania. *Reg. Stud. Mar. Sci.* **2016**, *7*, 72–80, doi:10.1016/j.rsma.2016.05.014.
36. Ahmed, Q.; Bat, L.; Yousuf, F.; Mohammad Ali, Q.; Nazim, K. Accumulation of Heavy Metals (Fe, Mn, Cu, Zn, Ni, Pb, Cd and Cr) in Tissues of Narrow-Barred Spanish Mackerel (Family-Scombridae) Fish Marketed by Karachi Fish Harbor. *Open Biol. Sci. J.* **2015**, *1*, 20–28, doi:10.2174/2352633501501010020.
37. Gu, Y.-G.; Lin, Q.; Wang, X.-H.; Du, F.-Y.; Yu, Z.-L.; Huang, H.-H. Heavy Metal Concentrations in Wild Fishes Captured from the South China Sea and Associated Health Risks. *Mar. Pollut. Bull.* **2015**, *96*, 508–512, doi:10.1016/j.marpolbul.2015.04.022.
38. Safiur Rahman, M.; Solaiman Hossain, Md.; Ahmed, Md.K.; Akther, S.; Jolly, Y.N.; Akhter, S.; Jamiul Kabir, M.; Choudhury, T.R. Assessment of Heavy Metals Contamination in Selected Tropical Marine Fish Species in Bangladesh and Their Impact on Human Health. *Environ. Nanotechnol. Monit. Manag.* **2019**, *11*, 100210, doi:10.1016/j.enmm.2019.100210.
39. Nurul Izzah, A.; Wan Rozita, W.M.; Tengku Rozaina, T.M.; Cheong, Y.L.; Siti Fatimah; Nasriyah, C.H.; Nor Aini, A.; Rafiza, S.; Lokman, H.S. Fish Consumption Pattern among Adults of Different Ethnics in Peninsular Malaysia. *Food Nutr. Res.* **2016**, *60*, 32697, doi:10.3402/fnr.v60.32697.
40. Ahmed, Q.; Bat, L. Accumulations of Zn, Ni, B, Al, and Co in Megalaspis Cordyla from Fish Marketed by Karachi Fish Harbor of Pakistan. *Int. J. Fish. Aquat. Stud.* **2015**, *2*, 204–207.
41. Janadeleh, H.; Jahangiri, S. Risk Assessment and Heavy Metal Contamination in Fish (Otolithes Ruber) and Sediments in Persian Gulf. *J. Community Health Res.* **2016**, *5*, 169–181.
42. Kamaruzzaman, Y.; Rina, Z.; John, B.A.; Jalal, K.C.A. Heavy Metal Accumulation in Commercially Important Fishes of South West Malaysian Coast. *Res J Env. Sci* **2011**, *5*, 595–602, doi:10.3923/rjes.2011.595.602.
43. Paudel, P.; Pokhrel, B.; Kafle, B.; Gyawali, R. Analysis of Heavy Metals in Some Commercially Important Fishes of Kathmandu Valley, Nepal. *Int. Food Res. J.* **2016**, *23*, 1005–1011.
